# Deep Learning Enables Spatial Mapping of the Mosaic Microenvironment of Myeloma Bone Marrow Trephine Biopsies

**DOI:** 10.1158/0008-5472.CAN-22-2654

**Published:** 2024-01-11

**Authors:** Yeman Brhane Hagos, Catherine S.Y. Lecat, Dominic Patel, Anna Mikolajczak, Simon P. Castillo, Emma J. Lyon, Kane Foster, Thien-An Tran, Lydia S.H. Lee, Manuel Rodriguez-Justo, Kwee L. Yong, Yinyin Yuan

**Affiliations:** 1 Centre for Evolution and Cancer and Division of Molecular Pathology, The Institute of Cancer Research, London, United Kingdom.; 2 Research Department of Haematology, University College London Cancer Institute, London, United Kingdom.; 3 Research Department of Pathology, University College London Cancer Institute, London, United Kingdom.; 4 Centre for Molecular Pathology, Royal Marsden Hospital, London, United Kingdom.

## Abstract

Bone marrow trephine biopsy is crucial for the diagnosis of multiple myeloma. However, the complexity of bone marrow cellular, morphologic, and spatial architecture preserved in trephine samples hinders comprehensive evaluation. To dissect the diverse cellular communities and mosaic tissue habitats, we developed a superpixel-inspired deep learning method (MoSaicNet) that adapts to complex tissue architectures and a cell imbalance aware deep learning pipeline (AwareNet) to enable accurate detection and classification of rare cell types in multiplex immunohistochemistry images. MoSaicNet and AwareNet achieved an AUC of >0.98 for tissue and cellular classification on separate test datasets. Application of MoSaicNet and AwareNet enabled investigation of bone heterogeneity and thickness as well as spatial histology analysis of bone marrow trephine samples from monoclonal gammopathies of undetermined significance (MGUS) and from paired newly diagnosed and posttreatment multiple myeloma. The most significant difference between MGUS and newly diagnosed multiple myeloma (NDMM) samples was not related to cell density but to spatial heterogeneity, with reduced spatial proximity of BLIMP1^+^ tumor cells to CD8^+^ cells in MGUS compared with NDMM samples. Following treatment of patients with multiple myeloma, there was a reduction in the density of BLIMP1^+^ tumor cells, effector CD8^+^ T cells, and regulatory T cells, indicative of an altered immune microenvironment. Finally, bone heterogeneity decreased following treatment of patients with multiple myeloma. In summary, deep learning–based spatial mapping of bone marrow trephine biopsies can provide insights into the cellular topography of the myeloma marrow microenvironment and complement aspirate-based techniques.

**Significance:**

Spatial analysis of bone marrow trephine biopsies using histology, deep learning, and tailored algorithms reveals the bone marrow architectural heterogeneity and evolution during myeloma progression and treatment.

## Graphical Abstract

**Figure:**
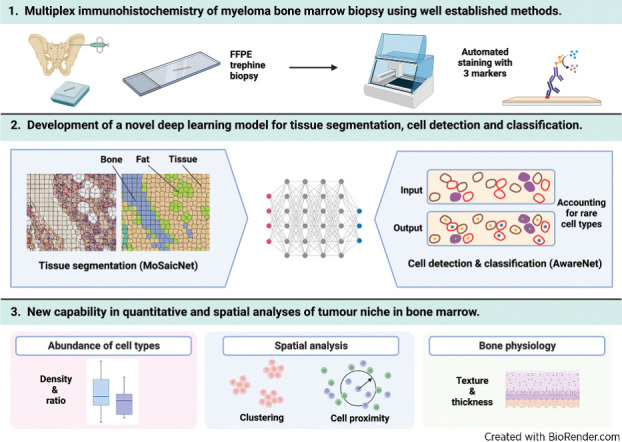

## Introduction

Multiple myeloma is an incurable hematologic malignancy characterized by the uncontrolled proliferation of abnormal plasma cells in the bone marrow (BM; refs. 1, 2, 3). According to the International Myeloma Working Group (IMWG), the current diagnosis of multiple myeloma is based on the demonstration of clonal neoplastic plasma cells and organ dysfunction, of which the most common is bone destruction, which is typically investigated by BM aspirate, trephine biopsy samples, and whole-body noninvasive imaging (4).

Increasingly, there is growing appreciation that myeloma is not driven by malignant plasma cells in isolation, but tumor growth is accompanied by global immune dysregulation in multiple myeloma (5, 6). These include impaired T-cell effector function (7) and antigen presentation (8) and an increase in suppressor cells such as regulatory T cells (Treg; refs. 9, 10, 11). Our previous work showed that patients with multiple myeloma who had high Tregs had shorter progression-free survival (11). In addition, analysis of CD4^+^ and CD8^+^ effectors revealed that a low CD4^+^ effector to Tregs ratio was an independent predictor of early relapse (11). However, these studies were based on multiple myeloma blood/BM aspirates or multiple myeloma cell lines employing flow cytometry and gene expression analysis, and not using biopsies that preserve the architecture of the BM. Therefore, the spatial relationship between BM cell types in multiple myeloma has not yet been studied.

Deep learning methods, specifically convolutional neural networks (CNN), have been shown to accurately identify complex visual patterns in histopathology images without handcrafted features (12, 13). This offers a unique opportunity to harness the cellular and noncellular mosaic spatial ecology of BM (12, 14). However, the unique tissue integrity and morphology of BM trephine samples are very different from those of solid tumors due to its specialized sampling process and its requirement for decalcification (Supplementary Fig. S1A). The BM also has a highly organized structure, being a specialized hemopoietic and immunologic organ. Thus, the BM is one of the priming sites of T cells and contains both rare and abundant cell types (Supplementary Fig. S1B; ref. 15); the spatial context of cell-to-cell interactions is likely to be crucially important in the development of immunity. Deep learning methods are often sensitive to the biases in the data unless carefully designed. Thus, there are new challenges in the development of reliable automated analysis for BM trephine samples due to possible biases in cell abundance and tissue architecture complexity.

In this study, we propose new deep learning–based image analysis pipelines addressing these challenges: (i) to dissect the mosaic tissue microenvironment of BM trephine samples (MoSaicNet) and accurately identify immune T and multiple myeloma plasma cells (AwareNet) on multiplex immunohistochemistry (MIHC) images; (ii) to harness the morphologic features of bone trabeculae in monoclonal gammopathies of undetermined significance (MGUS), diagnostic, and posttreatment multiple myeloma facilitating new understanding of bone physiology; (iii) to analyze cell density, infiltration pattern, and spatial topography of immune T and multiple myeloma plasma cells facilitating understanding of the cellular topography in the BM niche of MGUS, diagnostic and posttreatment multiple myeloma samples.

## Materials and Methods

### Patients studied

All patients were managed at University College London Hospital (UCLH). BM trephine biopsies from two cohorts of patients were extracted: 11 patients with MGUS and 14 patients with multiple myeloma. Two patient samples from the MGUS group and four patient samples from the multiple myeloma group were excluded because of suboptimal tissue samples (small areas of hematopoietic tissue), leaving 9 patients with MGUS and 10 patients with multiple myeloma included in this study. For the second group, we studied patients with newly diagnosed multiple myeloma (NDMM) prior to treatment initiation and also posttreatment, when BM biopsies were taken at 100 days following autologous stem cell transplant (ASCT). All patients provided written informed consent for this project. Ethical approval was granted by the Health Research Authority, UK (Research ethics committee reference: 07/Q0502/17).

Patient characteristics for the MGUS group are shown in Table 1. The median age was 61 years, and 56% were male. The majority had IgG MGUS (56%), 3 had IgA MGUS (33%), and 1 had kappa light chain MGUS (11%). Five patients (56%) were deemed to have a low risk of multiple myeloma progression, while 2 (22%) had intermediate risk, and 2 (22%) had a high risk (16).

**Table 1.** Patient characteristics: MGUS.

Patient characteristics (*n* = 9)	Patient no. (%)
Age at diagnosis	
Median (range)	61 (54–89)
Gender	
Male	5 (56)
Immunoglobulin (Ig) isotype	
IgG	5 (56)
IgA	3 (33)
Light chains only	1 (11)
Light chain isotype	
Kappa	5 (56)
Lambda	3 (33)
Polytypic	1 (11)
IMWG cytogenetics risk	
Standard risk	5 (56)
High risk	1 (11)
Unknown	3 (33)
Risk categories for progression to multiple myeloma	
Low	5 (56)
Intermediate	2 (22)
High	2 (22)

The characteristics of the 10 patients in the multiple myeloma group are described in Table 2. The median age at multiple myeloma diagnosis was 56 years, consistent with an age group that would usually proceed with treatment following induction therapy. Six (60%) patients were male, 5 had IgG disease (50%), and half had standard cytogenetic risk by IMWG criteria. Four patients (40%) had International Staging System (ISS) stage I disease, 5 (50%) had stage II, and 1 (10%) had stage III (17). All patients received combination induction therapy with a proteasome inhibitor, cyclophosphamide and dexamethasone, followed by melphalan 200 mg/m^2^ as a conditioning regimen prior to ASCT.

**Table 2.** Patient characteristics: paired diagnostic and posttreatment samples.

Patient characteristics (*n* = 10)	Patient no. (%)
Age at diagnosis	
Median (range)	56 (53–63)
Gender	
Male	6 (60)
Immunoglobulin (Ig) isotype	
IgG	5 (50)
IgA	2 (20)
Light chains only	3 (30)
Light chain isotype	
Kappa	7 (70)
Lambda	3 (30)
IMWG cytogenetics risk	
Standard risk	5 (50)
High risk	5 (50)
IMWG ISS staging	
I	4 (40)
II	5 (50)
III	1 (10)
PC % in diagnostic BM biopsy	
Median (range)	70% (13–80)
Line of therapy at treatment	
1	10 (100)
Induction therapy	
KCD	10 (100)
PC % at D100 BM biopsy posttreatment	
Median (range)	0.5% (0–10)

Abbreviations: C, cyclophosphamide; D, dexamethasone; D100, day 100; K, carfilzomib; PC, plasma cell.

### Tissue processing

BM samples were collected and processed as per International Council for Standardization in Hematology (ICSH) guidelines (18). They were first fixed in neutral buffered formalin and then decalcified with formic acid. After decalcification, biopsy specimens were embedded in paraffin wax and cut on a microtome at 2–3 μm. Serial sections were cut and mounted on glass slides.

### MIHC panel selection

Immune T cells play an active role in the disease's development and progression in multiple myeloma. In this study, we aimed to analyze the density and the spatial topography of immune T and multiple myeloma tumor cells in BM trephine biopsies. We chose CD4 and CD8 to label effector T cells, FOXP3 to represent Tregs (19), and BLIMP1 to stain multiple myeloma tumor cells (20–22).

The MIHC staining was performed using the fully automated Leica Bond RX^m^ stainer. Each slide was serially stained to identify three different antigens using different membranous or nuclear stains. The details of antibodies used are in Supplementary Table S1. Two MIHC multiplex panels were used in this study. Panel 1 included T-cell markers CD4 and CD8, as well as FOXP3, a transcription factor specifically expressed by CD4^+^ Tregs. Panel 2 comprised CD4, CD8, and BLIMP1. BLIMP1 is a nuclear stain and therefore allowed clear visualization when combined with CD4 and CD8 membranous stains. Staining protocols can be found in (Supplementary Tables S2 and S3). Stained slides were then scanned using the Hamamatsu Nanozoomer s360 scanner and analyzed by the deep learning models.

### Preprocessing of whole slide images

The MIHC whole slide images (WSI) were scanned at 40× magnification with a pixel resolution of 0.23 μm/pixel. A representative image has a 40,000×40,000 pixel size at ×40 magnification. For efficient image processing, the images were downscaled to ×20 magnification and divided into 2,000×2,000 pixel “tiles”.

### MoSaicNet: segmenting BM trephine components using deep learning and superpixel

**Figure 1.**
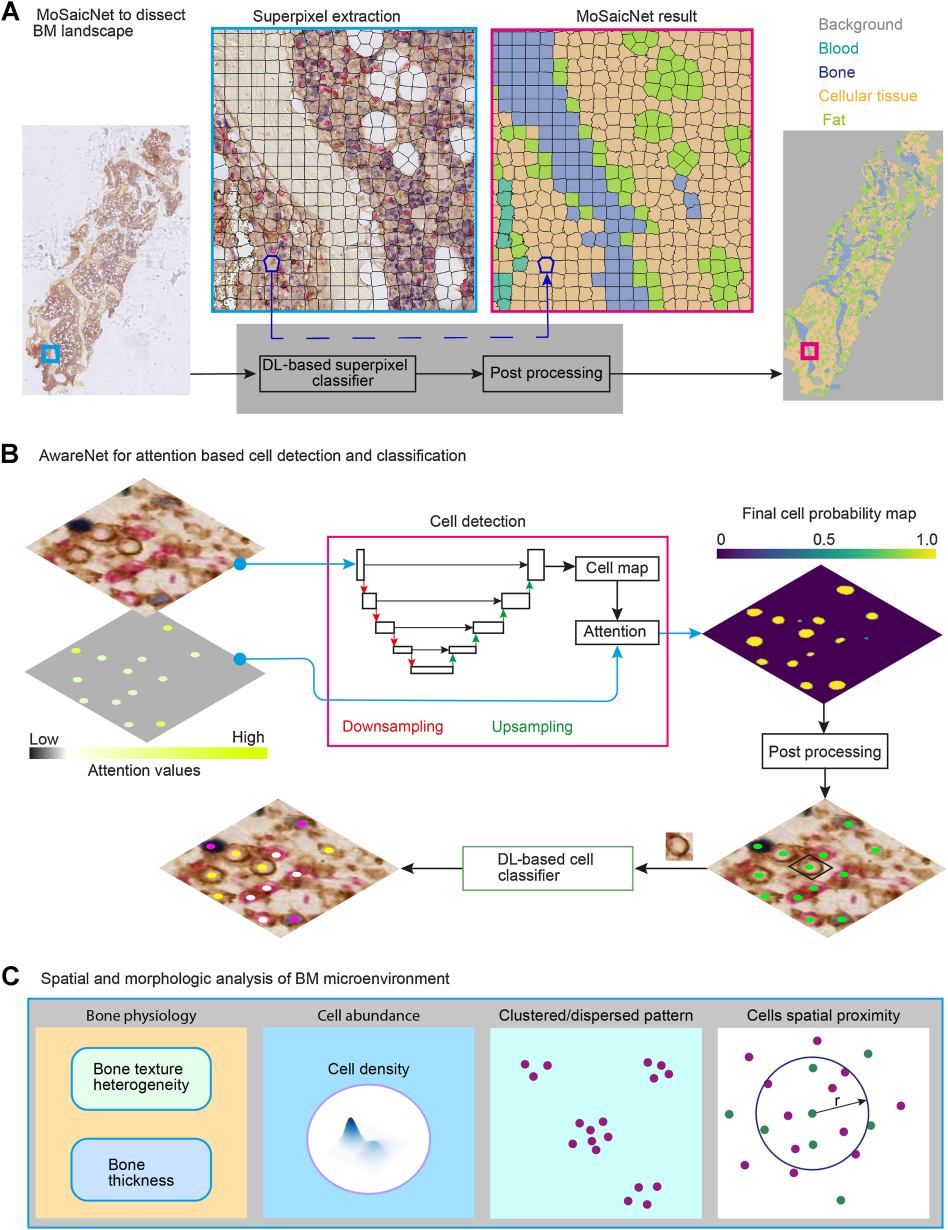
Overview of computational deep learning and image processing pipelines for BM MIHC images: **A,** MoSaicNet pipeline. The polygons (black) indicate superpixels. MoSaicNet dissects a tissue section into bone, blood, fat, and cellular tissue regions (Supplementary Materials and Methods). **B,** AwareNet for attention-based cell detection and classification (Supplementary Materials and Methods). The attention image pixel values were generated from the abundance of cell types. An attention image was applied to the objective function during model parameter optimization to regularize the algorithm by assigning high attention to rare cell types. The cell detection algorithm generates a cell probability map. A postprocessing algorithm was developed to find the cell nucleus center, (*x*, *y*) location, from the probability map (Supplementary Materials and Methods). A patch centered on each cell was extracted and fed to deep learning (DL)-based classifier to infer its class. **C,** Spatial and morphologic analysis of BM trephine samples. Bone texture and structural heterogeneity were investigated using an autoencoder-based machine learning method (Supplementary Materials and Methods). We used spatial proximity analysis to study the spatial relations of cells. *r*, radius. Cell density refers to the number of cells per unit of tissue area.

The digital image of the BM trephine is a mosaic landscape of blood, bone, cellular tissue, and fat region (Supplementary Fig. S1A). To automatically segment these regions, we developed MoSaicNet (Morphological Analysis with **S**uperpixel-based Habitat Detection Network; Fig. 1A). MoSaicNet contains superpixel extraction and a CNN-based superpixel classifier.

### MoSaicNet training and validation data preparation

To train, validate, and test MoSaicNet, we collected 260 regions of interest from 19 samples (Supplementary Table S4) annotated by expert pathologists (Supplementary Fig. S2A) from the different regions of the images. The training (47%), validation (31%), and testing (22%) split was randomly done at the patient level. These annotated regions were extracted from the WSIs and divided into superpixels using the simple linear iterative clustering (SLIC) superpixels algorithm (Fig. 1A; ref. 23). SLIC groups neighboring pixels with similar pixel intensity into one superpixel. The shape of the superpixels is controlled by the compactness (*C*) parameter of the SLIC algorithm. The number of superpixels depends on the size of the images and the parameter *k* (Eq. A; refs. 23, 24). The parameters *C* and *k* are set by a user to ensure superpixels are capturing homogeneous pixels and bounding to region boundaries in the image under consideration depending on the scenario (23, 24). The number of superpixels (*n*) was computed using Eq. A.

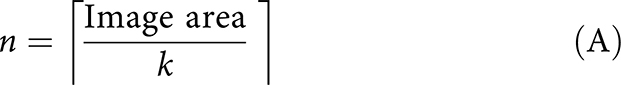


Upon visual assessment, superpixels with *k* = 2,000 and *C* = 30 best adhere to the boundaries of tissue and fat regions. This resulted in about 40×40 pixel (18.4 μm × 18.4 μm) sized superpixel regions (Fig. 1A). After applying SLIC, we generated 69, 884 superpixels from the 260 regions (Supplementary Table S5). These superpixels belonged to four classes: blood, bone, fat, and cellular tissue. Each superpixel was assigned a class of the region it belongs. We implemented and trained a custom-designed convolutional neural network to automatically classify these superpixel regions (Supplementary Materials and Methods).

### AwareNet: attention-based deep convolutional network for cell detection and classification

#### Single-cell annotation

To train, validate, and test our proposed deep learning–based single-cell detection and classification models, we first collected 8,004 single-cell dot annotations on 11 samples by expert pathologists (Supplementary Fig. S2A), using a web-based annotation tool developed in our lab (not published). The annotations belonged to three classes: CD8^+^ (*n* = 5103), FOXP3^−^CD4^+^ (*n* = 2381), and FOXP3^+^CD4^+^ (*n* = 518). We identified FOXP3^+^ cells as rare because they represented only 6.5% of all annotated cells, despite histopathologists actively looking for them in the whole tissue instead of only regions of interest. The training (46%), validation (27%), and test (27%) split was done randomly at the patient level to ensure that cells from the same patients are not included in different categories (Supplementary Table S6).

#### Cell detection and classification

To automatically localize cells in MIHC images, we developed AwareNet (Fig. 1B). AwareNet is a deep learning method designed to give high attention to rare cell types such as FOXP3^+^CD4^+^ cells in the case of BM trephine samples. During model training, the attention score was inferred from the relative abundance of each cell type in the training data. A rare cell type was given a larger attention score. The mathematical formulation of attention image generation and usage during model training is detailed in ref. 25.

AwareNet generates a predicted cell nucleus center probability map image (Fig. 1B) from which the spatial coordinates of the center of the cell's nucleus are computed (detailed in Supplementary Materials and Methods). To identify the type of the detected cell, we extracted a 28×28×3 patch centered on the cell nucleus (Fig. 1B) and applied a custom-designed CNN classifier (25).

#### Cell density

Cell density is measured as the number of cells per unit of tissue area (μm^2^). Suppose a given tissue section has *N* cells and cellular tissue area of *A_T_*, cell density is computed using Eq. B.

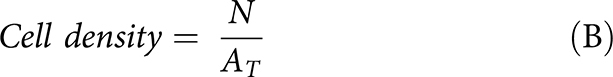


#### Cell proximity analysis

We investigated the spatial proximity of a pair of cell types (e.g., BLIMP1^+^ multiple myeloma plasma cells and CD8^+^ T cells) within the BM microenvironment as follows (Fig. 1C). Consider a tissue section that contains *k* number of type A cells located at {*a_i_*, *i* ∈ {1, 2, 3, …, *k*}} and *m* number of type B cells located at {*b_j_*, *j* ∈ {1, 2, 3, …, *m*}}. Each cell has an (*x*, *y*) position. The number of type B cells within a distance ***r*** from type A cell was computed using Eq. C, i and C, ii.

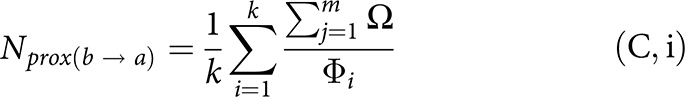

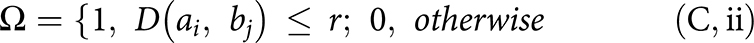


where *D* is the Euclidean distance function for two cells, *a_i_* and *b_j_*. Ф_i_ is a normalizing factor, which is the total number of cells (all types) within *r* distance from *a_i_*. In BM trephine samples, there is a huge variation in the tissue architecture caused by the prevalence of noncellular regions such as bone and fat regions (Supplementary Fig. S1A). Moreover, in single cell–based spatial analysis, the density of cells could be a confounding factor. Incorporating Ф*_i_* corrects these factors.

#### Validation cohort

BM trephine samples from a separate patient cohort were used to validate this deep learning pipeline. This cohort consisted of nine NDMM pretreatment and posttreatment BM samples. Patient characteristics can be found in Supplementary Table S7. These were collected from seven different U.K. hospitals (one from UCLH, one Kent & Canterbury Hospital, two Sunderland Royal Hospital, one Warwick Hospital, one Calderdale Royal Hospital, two Ninewells Hospital, one Huddersfield Royal Infirmary) and were stained with MIHC panel 2 (CD4, CD8, and BLIMP1) using the same staining protocol. A different autostainer of the same model was used. WSIs were scanned and underwent color normalization (Supplementary Materials and Methods) before analysis to adjust for tissue processing and staining variations.

### Bone density similarity and heterogeneity

To learn the low-dimensional representation of bone superpixels, we custom-designed a convolutional autoencoder (Supplementary Materials and Methods, Supplementary Fig. S2B). For ease of visualization and applying unsupervised clustering algorithms on the representation of bone superpixels, we applied Uniform Manifold Approximation and Projection (UMAP) dimensionality reduction.

Then, we applied a clustering algorithm to divide the latent representation space into smaller regions. Kmeans and Gaussian mixture models (GMM) are the most commonly used clustering algorithms. We applied GMM to detect bone superpixel clusters in the embedding space due to its flexibility to cluster shapes (26). To determine the number of clusters, we used the Akaike information criterion (AIC) and the Bayesian information criterion (BIC). We used the GMM algorithm and its built-in AIC and BIC methods from the Scikit-Learn python package (27). A cluster contains superpixels with similar bone density/texture. The clustering enabled us to identify artefact bone superpixels with input from an expert pathologist (M. Rodriguez-Justo). These clusters were excluded from further analysis.

To quantify the heterogeneity (H) of bone texture within a slide, we computed the maximum variance (Var) of the latent representations of all superpixels within the slide using Eq. D.

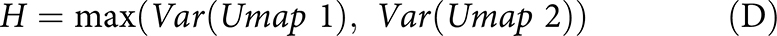


### Automated machine learning algorithm to quantify bone thickness

**Figure 2.**
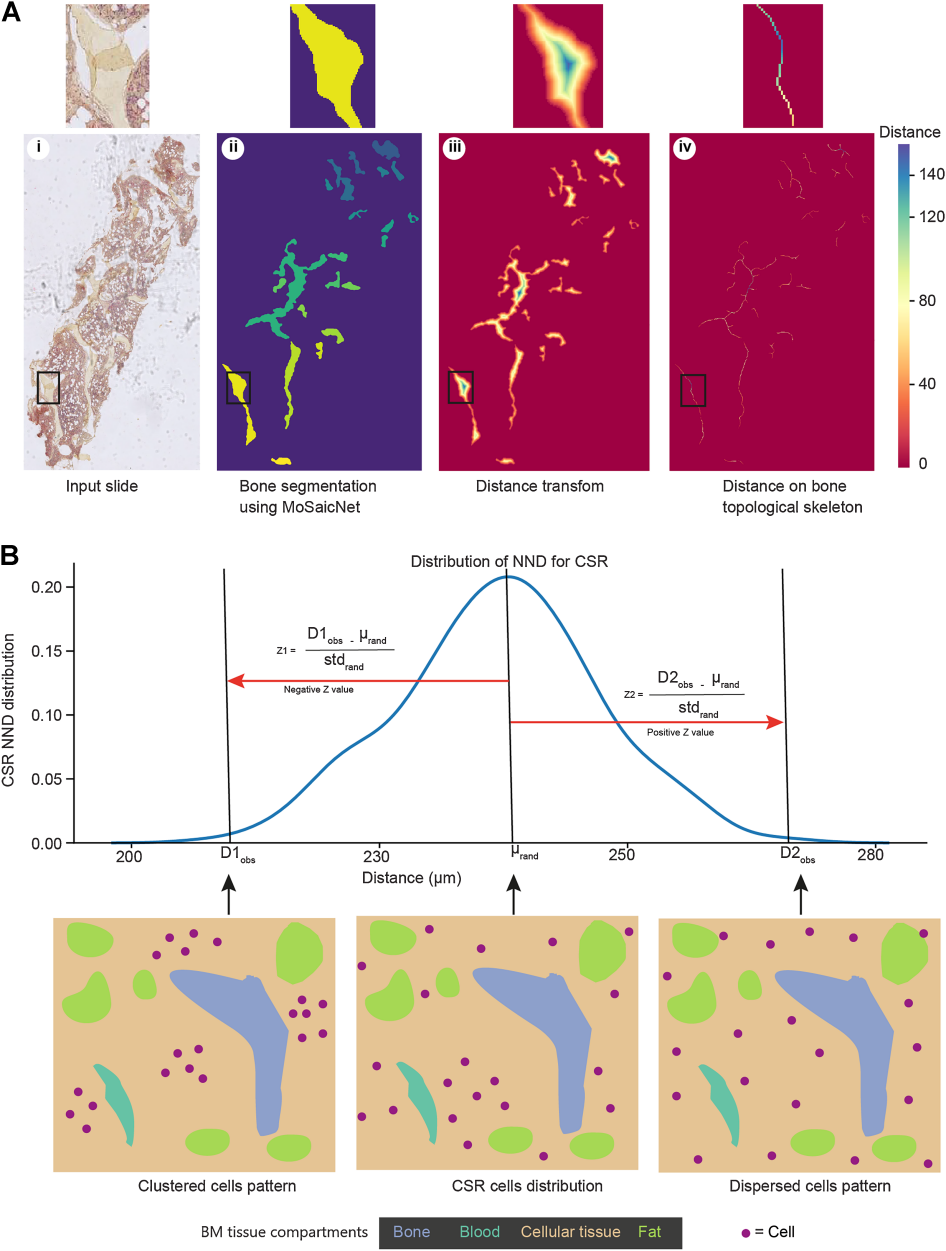
Computational methods for bone thickness analysis and cell infiltration patterns: **A,** Image analysis to estimate bone thickness (Supplementary Materials and Methods). Using the same BM sample image as Fig. 1A, the bone segmentation (ii) is an output of MoSaicNet (Supplementary Materials and Methods), and each bone is displayed in a different color. The color bar shows the pixel intensity of the image in iii and iv. The pixel intensity on the skeleton indicates half of the bone thickness (Supplementary Materials and Methods). **B,** Cell infiltration pattern analysis using NND and the null hypothesis of CSR (Supplementary Materials and Methods). Z < −1.96, Z > 1.96, and −1.96 ≤ Z ≤ 1.96 indicate a clustered, dispersed, and random distribution of observed cells, respectively. std, standard deviation; μ, mean NND of CSR.

The proposed method to quantify bone thickness is shown in Fig. 2A. We extracted the bone regions from the output of MoSaicNet. To compute bone thickness for a given bone (*B*), first, we applied distance (28), and medial axis transforms (29) as shown in Fig. 2A. The distance transform (DT) computes the minimum distance from bone pixels to non-bone pixels. The medial axis transform (MAT) generates the topological skeleton of the bone, a series of bone pixels that have more than one closest equidistant non-bone pixel. The bone thickness (B_thickness_) for a given tissue sample was computed as the mean of the mean thicknesses of all bones within the sample using Eq. E.

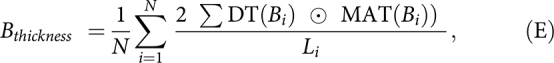

where *N* is the number of bones in the sample, and ⊙ is elementwise matrix multiplication. *L_i_* is the length (number of pixels) of the skeleton of the *i*^th^ bone, *B_i_*. The distance values on the medial axis of the bone are half the thickness of the bone across its length. Thus, to get the total bone thickness, the distance was multiplied by 2, as shown in Eq. E.

### Spatial analysis

To quantify the degree of clustering or dispersion of cells in BM trephine samples, we used the concept of nearest neighbor distance (NND) and the null hypothesis to identify the infiltration pattern of cells (Supplementary Materials and Methods). NND is the distance from a spatial point to its closest neighbor. Under the null hypothesis, which is complete spatial randomness (CSR), the distribution of NND is normal. (Fig. 2B). We computed the Z-score to measure the difference between the NND for random distribution of cells and the NND of observed cells pattern. Z < −1.96, Z > 1.96, and −1.96 ≤ Z ≤ 1.96 indicate a clustered, dispersed and random distribution of observed cells, respectively.

### Statistical analysis

All statistical analyses were carried out using the Python programming language. All correlation values were measured using the nonparametric Spearman test. The *P* values were computed using a two-sided unpaired (for MGUS vs. NDMM) or paired (for NDMM vs. posttreatment), nonparametric Wilcoxon method, considering *P* < 0.05 as significant. Benjamini–Hochberg (BH) correction was applied in the case of multiple comparisons to maintain the experiment-wise type I error rate at 0.05.

### Code and data availability

All methods and analyses were implemented in Python. The tested implementation of methods listed above can be found on this Code Ocean link (https://codeocean.com/capsule/0863619/tree/v1) along with documentation explaining how to run the different algorithms. A Docker file containing all the dependencies and a test .ndpi WSI is also included in Code Ocean repository. This repository contains an end-to-end analysis of WSI comprising of Tiling, superpixel-based tissue classification, cell detection, cell classification, cell counting, bone thickness quantification, and cell proximity quantification. In Code Ocean, at test WSI is uploaded and pressing the “Reproducible Run” button at the top right corner will automatically perform the above listed tasks and output will be saved in results folder. The code runs on both local and high-performance clusters using the Docker container. All raw data are available from the corresponding authors upon request.



## Results

### Computational and spatial analysis

Unlike solid tumors, BM trephine sections consist of isolating structural elements over different spatial scales, reflecting a mix of cellular communities and mosaic habitats. To dissect this complex tissue landscape and detect rare cells in MIHC (Supplementary Fig. S1), we specifically designed two deep learning methods, MoSaicNet to dissect the mosaic landscape of BM tissue (Fig. 1A) and AwareNet to detect and classify cells (Fig. 1B). First, to dissect the multiple myeloma tissue into blood, bone, fat, and cellular tissue patches/habitats, a superpixel-based deep learning method was designed to capture the complex landscape (Fig. 1A). To train and validate MoSaicNet, we collected expert segmentation annotations for 260 regions, which resulted in 69,884 superpixels (Supplementary Materials and Methods; Supplementary Tables S4 and S5). Subsequently, we were able to quantify the amount of cellular tissue, which served as an important quality control parameter, to determine whether a slide would be considered for further analysis. With the help of our pathologist, the tissue area threshold was set to 1.1 × 10^6^ μm^2^. Sections with cellular tissue area less than this threshold were excluded from analysis.

To optimally detect and classify cells within BM trephine samples, that contain both rare (e.g., FOXP3^+^CD4^+^) and abundant cells (Supplementary Fig. S1B). To optimally detect and classify cells within BM trephine samples that contain both rare (e.g., FOXP3^+^CD4^+^) and abundant cells (Supplementary Fig. S1B), we developed AwareNet (25).

Subsequently, we analyzed the BM spatial microenvironment in terms of cell density, cell ratio, cell spatial proximity and clustering, and bone physiology in terms of bone density/texture heterogeneity, and bone thickness (Fig. 1C; Supplementary Materials and Methods).

### High accuracy of MoSaicNet classification model

**Figure 3.**
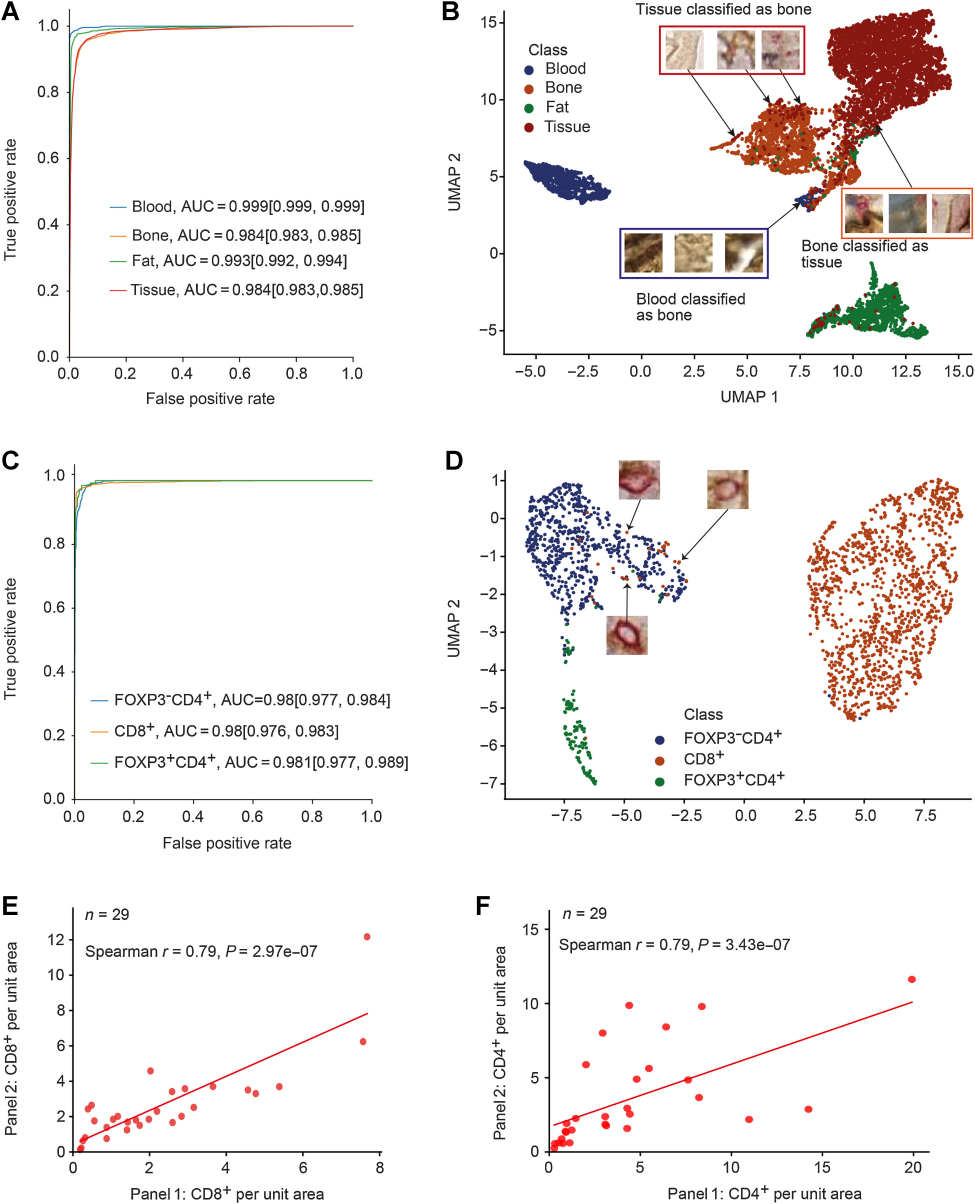
Performance evaluation of MoSaicNet and AwareNet deep learning models: **A**, The ROC curves and AUC values of the MoSaicNet superpixel classifier. The values in brackets indicate the 95% CI. **B,** Two-dimensional mapping of superpixels using MoSaicNet learned 200-dimensional features after dimensionality reduction by UMAP. **C,** The ROC curves and AUC values of single-cell classifier model on separately held test data. The values in brackets indicate the 95% CI. **D,** UMAP features visualization of deep learned features by AwareNet single-cell classifier CNN. **E** and **F,** Validation of AwareNet model using correlation of density of CD8^+^ (**E**) and CD4^+^ (**F**) cells in panel 1 and panel 2.

To evaluate the performance of the MoSaicNet classification model, we used 9,330 superpixels extracted from separately held manually annotated samples (Supplementary Table S5). The superpixels belonged to the blood, bone, fat, and cellular tissue classes. To measure the classifier's performance, we used accuracy, AUC, precision, recall, and F1-score (Supplementary Materials and Methods). Taking all classes together, the superpixel classifier model achieved an AUC value of 0.99, 95% confidence interval (CI, 0.989–0.991; Supplementary Table S8). Moreover, for each class, the bootstrap mean AUC was >0.984 for all the classes (Fig. 3A; Supplementary Table S8). The overall accuracy (unweighted) was 0.937, 95% CI (0.935–0.94).

Out of the 9,330 superpixels, 585 superpixels were misclassified. Out of the 585 misclassified superpixels, 208 tissue superpixels were misclassified as bone, and 122 bone superpixel patches were misclassified as tissue (Supplementary Fig. S3A). This was also evident in the lower precision value for bone class [0.88, 95% CI (0.87–0.89)], lower recall value for bone class [0.933, 95% CI (0.93–0.94)], and lower recall value for cellular tissue class [0.932, 95% CI (0.93–0.94)] (Supplementary Table S8) compared with other classes. Moreover, 88 tissue superpixels and 29 bone superpixels were misclassified as a fat class, and the precision score for the fat class was 0.933, 95% CI (0.93–0.94; Supplementary Table S8). Areas under precision-recall curves (AUC-PR) were >0.95 across all classes (Supplementary Fig. S3B). A mean F1-score of 0.91 was obtained for the bone class, and for the other classes, the mean F1-score was 0.943. Taking all classes together, an F1-score of 0.94, 95% CI (0.935–0.945) was obtained (Supplementary Table S8).

Most of the tissue superpixels misclassified as bone were superpixels with poor tissue quality, noncellular regions, and bone-bordering areas (Fig. 3B). Most of the 122 bone superpixels that were misclassified as tissue were a result of background staining of the bordering area (Fig. 3B).

### Detecting rare cell types with AwareNet

To evaluate the performance of AwareNet, we measured precision, recall, and F1-score on separately held 2,131 test single-cell annotations. AwareNet achieved an F1-score of 0.78, a 2% increase compared with U-net (30) and a 1% increase compared with CONCORDe-Net (13). In particular, AwareNet excels in detecting FOXP3^+^CD4^+^ cells, which are rare in BM trephines (representing ∼7% of the training data; ref. 25).

Taking all three classes together, the single-cell classifier model of AwareNet achieved an AUC value of 0.98, 95% CI (0.977–0.984; Supplementary Table S9). Moreover, for each class, the mean bootstrap AUC value was >0.98, with a minimum AUC 95% CI lower bound of 0.976 for the CD8^+^ class (Supplementary Table S9; Fig. 3C). The overall accuracy (unweighted) was 0.965, 95% CI (0.962–0.969). Out of the 2,131 cells, 74 cells were misclassified (Supplementary Fig. S3C). A total of 11 cells out of 135 FOXP3^+^CD4^+^ cells were misclassified as FOXP3^−^CD4^+^ cells, and 12 FOXP3^−^CD4^+^ cells were misclassified as FOXP3^+^CD4^+^ cells (Supplementary Fig. S3C). This resulted in precision [0.857, 95% CI (0.83–0.89)], recall [0.92, 95% CI (0.9–0.94)], and F1-score [0.887, 95% CI (0.87–0.91)] for the FOXP3^+^CD4^+^ class (Supplementary Table S9). Precision-recall curves are displayed in Supplementary Fig. S3D and the AUC-PR of the rarer cell type, FOXP3^+^CD4^+^, was 0.82. For the FOXP3^−^CD4^+^ and CD8^+^ class, the F1-score was 0.956, 95% CI (0.95–0.96), and 0.98, 95% CI (0.98–0.98), respectively (Supplementary Table S9). Moreover, when all classes were combined, the classifier obtained an F1-score of 0.941, 95% CI (0.93–0.95; Supplementary Table S9). The Matthew correlation coefficient was 0.93 for this panel.

UMAP-based inspection of the misclassified FOXP3^−^CD4^+^ and CD8^+^ cells revealed that these cells were mainly cells coexpressing both CD8 and CD4 proteins (Fig. 3D; Supplementary Materials and Methods). These rare cell types have been found in follicular lymphoma (31) and urological cancers (32) but, to the best of our knowledge, they have not been studied in myeloma.

AwareNet was trained on single-cell data from CD4/CD8/FOXP3 panel data and directly applied to both panels, CD4/CD8/FOXP3 and CD4/CD8/BLIMP1. After applying the model to both panels, the numbers of CD8^+^ and CD4^+^ cells in both panels were significantly correlated (*r* = 0.79, *P* = 2.97 × 10^−7^ and *r* = 0.79, *P* = 3.43 × 10^−7^, Fig. 3E and F, respectively), validating the reliability of AwareNet. All cell frequencies from both panels detected by AwareNet can be found in Supplementary Table S10.

### MoSaicNet reveals changes in bone physiology posttreatment

**Figure 4.**
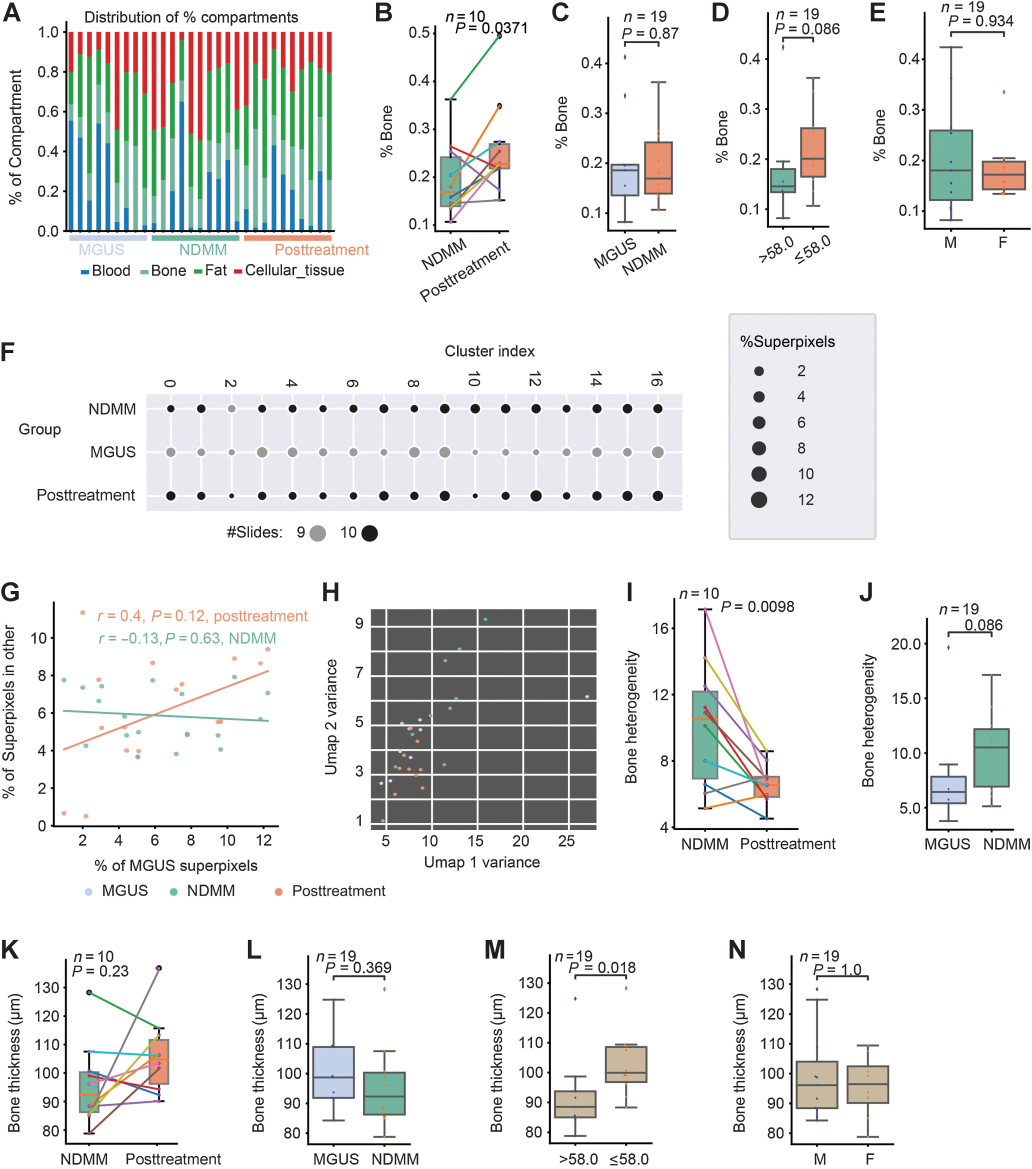
Studying bone physiology using MoSaicNet. **A**, Proportion of different compartments of BM trephine digital images. **B–E,** One stacked bar represents a sample. Box plots showing the difference in %bone between samples from NDMM and posttreatment (**B**), MGUS and NDMM (**C**), different age groups (median age = 58.0 years; **D**), and gender groups (**E**). **F,** Scatter plot showing the number of bone superpixels in 17 clusters from MGUS, NDMM, and posttreatment samples. The size of the dots represents the percentage of superpixels. The color represents the number of slides in each cluster. **G,** Correlation of percentage of superpixels in each cluster between different patient groups. A point represents a cluster. **H,** Scatter plot of slide-level heterogeneity of bone features measured by features variance (Supplementary Materials and Methods). A point represents a patient/slide. **I** and **J,** Box plots showing differences in bone density heterogeneity between NDMM and posttreatment (**I**), and between MGUS and NDMM (**J**). **K–N,** Box plots showing the difference in bone thickness between samples from NDMM and posttreatment (**K**), MGUS and NDMM (**L**), and different age groups (median age = 58.0 years; **M**) and gender (**N**).

Using MoSaicNet, we quantified the proportion (%) of blood, bone, fat, and cellular regions in all sections (Fig. 4A). In the myeloma group, posttreatment trephine samples contained a greater proportion of bone (%bone) when compared with diagnostic samples (*P* = 0.037; Fig. 4B). There was a trend of decrease in %bone with age (*P* = 0.086). There was, however, no difference in the %bone between MGUS and NDMM or between male and female patients (Fig. 4C–E). There was a trend of increase in %fat at posttreatment compared with diagnostic sample pair (*P* = 0.05; Supplementary Fig. S4A) but was not different between MGUS patients and patients with NDMM, nor between age or gender (Supplementary Fig. S4B–S4D).

To investigate the heterogeneity of bone structure in BM samples, we used a convolutional autoencoder to learn the embedding of 177,600 bone superpixels extracted from nine MGUS (27.8%), 10 NDMM (34.4%), and 10 posttreatment (37.8%) WSIs (Supplementary Materials and Methods). Bone superpixels were mapped into 32 feature vectors and clustered into 17 groups (Supplementary Materials and Methods; Fig. 4F; Supplementary Fig. S4E–S4G). On the basis of this grouping, there was a positive trend in the similarity of bone superpixels from MGUS to bone superpixels from posttreatment samples compared with bone superpixels from NDMM samples, even though this was not significant (*r* = 0.4, *P* = 0.12 and *r* = −0.13, *P* = 0.63, Fig. 4G).

We then asked whether the bone texture differed between the patient groups. The intrasample and intersample bone texture or density heterogeneity in NDMM was significantly higher at diagnosis compared with posttreatment (Supplementary Materials and Methods, *P* = 0.0098, Fig. 4H and I). Moreover, we observe a pattern of increased bone heterogeneity in NDMM samples compared with MGUS samples; however, this was not significant (*P* = 0.086, Fig. 4H and J). The bone heterogeneity was similar between MGUS and posttreatment samples (Fig. 4H and *P* = 0.87; Supplementary Fig. S4H).

Furthermore, to analyze bone thickness, we developed an automated image analysis algorithm (Supplementary Materials and Methods; Fig. 2A). The bone thickness of NDMM samples was similar to posttreatment samples (*P* = 0.23, Fig. 4K) and MGUS (*P* = 0.37, Fig. 4L). The bone thickness in patients ages ≤58 years (median) was significantly higher compared with that in patients ages >58 years (*P* = 0.018, Fig. 4M), without variation between gender (*P* = 1.0, Fig. 4N).

### Decreased FOXP3^+^CD4^+^ and BLIMP1^+^ cell density posttreatment

**Figure 5.**
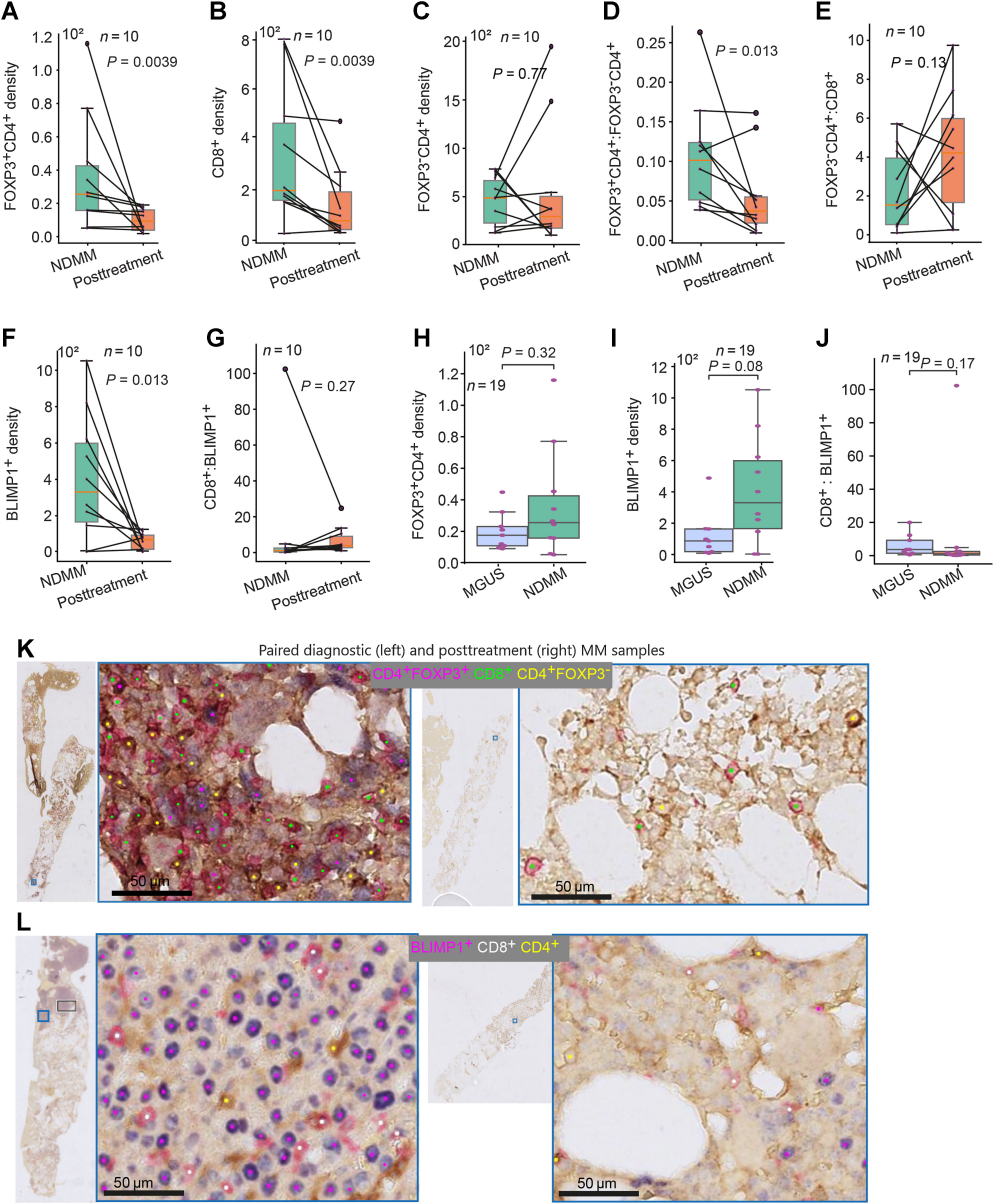
Density of immune T cells and plasma cells in MGUS, NDMM, and posttreatment samples. **A–G,** Box plots showing the difference in density of FOXP3^+^CD4^+^ (**A**), the density of CD8^+^ (**B**), the density of FOXP3^−^CD4^+^ (**C**), FOXP3^+^CD4^+^:FOXP3^−^CD4^+^ ratio (**D**), FOXP3^+^CD4^+^:CD8^+^ ratio (**E**), density of BLIMP1^+^ (**F**), and CD8^+^:BLIMP1^+^ ratio (**G**) between paired NDMM samples and posttreatment samples (*n* = 10 pairs). **H–J,** Box plot showing the difference in density of FOXP3^+^CD4^+^(**H**), the density of BLIMP1^+^ cells (**I**), and CD8^+^:BLIMP1^+^ cells (**J**) between MGUS and NDMM samples (*n* = 19). **K** and **L,** Sample images showing the reduction of the density of FOXP3^+^CD4^+^ and CD8^+^ cells (**K**) and BLIMP1^+^ cells (**L**) at posttreatment compared with paired NDMM samples. The cell density is presented per 1 mm^2^ tissue area.

When comparing cell density on the NDMM and posttreatment samples, we observed a decrease in both Tregs (FOXP3^+^CD4^+^), as well as CD8^+^ T cells following treatment (*P* = 0.0039 and *P* = 0.0039, respectively, Fig. 5A and B). However, FOXP3^−^CD4^+^ T-cell density did not change posttreatment (*P* = 0.77, Fig. 5C). The FOXP3^+^CD4^+^:FOXP3^−^CD4^+^ ratio is significantly reduced after ACST (*P* = 0.0137, Fig. 5D), largely due to the reduction in the density of FOXP3^+^CD4^+^ cells posttreatment. However, the FOXP3^−^CD4^+^:CD8^+^ ratio (CD4^+^ effector:CD8^+^ effector cells ratio) and the FOXP3^+^CD4^+^:CD8^+^ ratio were not different between the two timepoints (Fig. 5E; Supplementary Fig. S5A, respectively). We defined FOXP3^−^CD4^+^ cells as CD4^+^ effector T cells and CD8^+^ cells as CD8^+^ effector T cells. Tumor burden as measured by BLIMP1^+^ cells per unit area was significantly reduced posttreatment compared with the paired diagnostic samples (*P* = 0.0134; Fig. 5F). However, the CD8^+^:BLIMP1^+^ and CD4^+^:BLIMP1^+^ ratios were not significantly different between the diagnostic and posttreatment pairs (*P* = 0.275, Fig. 5G and *P* = 0.43, Supplementary Fig. S5B, respectively).

### Increased spatial proximity between BLIMP1^+^ cells and CD8^+^ cells in NDMM compared with MGUS

The density and ratio of CD8^+^, FOXP3^+^CD4^+^, and FOXP3^−^CD4^+^ cells were not significantly different between MGUS and NDMM (Fig. 5H; Supplementary Fig. S5C–S5G). There was a pattern of increase in BLIMP1^+^ cells density and BLIMP1^+^:CD4^+^ ratio in the NDMM sample compared with MGUS samples, though this was not significant (*P* = 0.08, Fig. 5I, and *P* = 0.08, Supplementary Fig. S5H, respectively). Furthermore, the ratio of the number of BLIMP1^+^ cells to CD8^+^ cells did not differ between MGUS and NDMM (*P* = 0.165, Fig. 5J). The density of FOXP3^+^CD4^+^ cells was significantly correlated with the density of BLIMP1^+^ cells in the posttreatment (*r* = 0.79, *P* = 0.006; Supplementary Fig. S5I) samples but not in MGUS and NDMM samples (*r* = 0.47, *P* = 0.205 and *r* = 0.20, *P* = 0.58; Supplementary Fig. S5I, respectively). Figure 5K and L are paired pretreatment and posttreatment BM examples that illustrate a reduction in FOXP3^+^CD4^+^, CD8^+^, and BLIMP1^+^ cell densities posttreatment.

**Figure 6.**
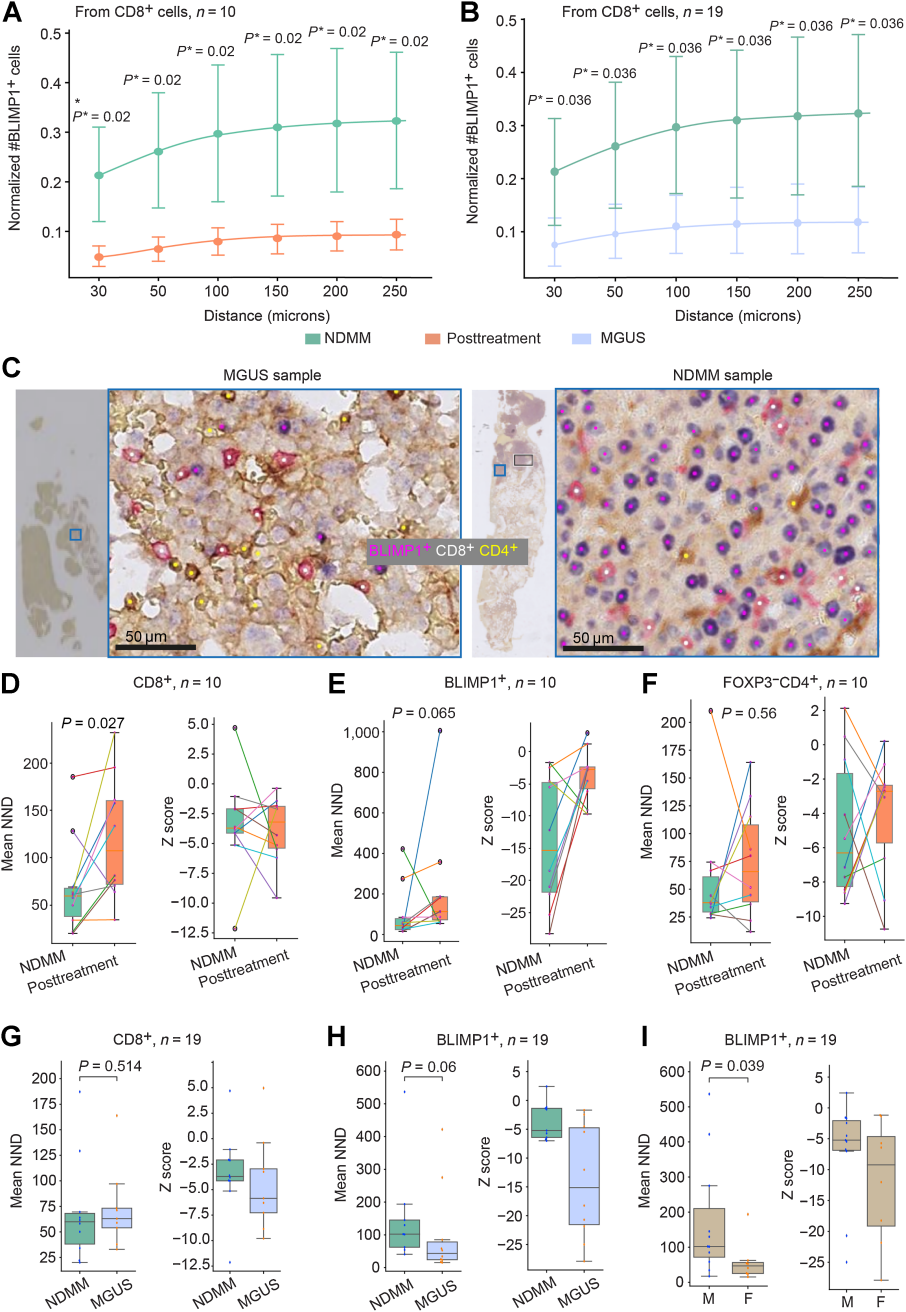
**A** and **B,** Spatial neighborhood of immune and tumor cells (**A** and **B**) and between MGUS and NDMM (**B**). The P* indicate *P* values after multiple testing correction using the BH method. The points represent the mean and the bars are 95% CIs, indicating uncertainty. **C,** Sample images showing an increased number of BLIMP1^+^ cells in the neighborhood with CD8^+^ on NDMM samples (NDMM example shown here is the same image as Fig. 5L) compared with MGUS samples. **D–I,** Clustered or dispersed pattern of immune and tumor cells in BM trephine sample. Box plots showing the difference in NND and Z score between NDMM and posttreatment for CD8^+^ cells (**D**), BLIMP1^+^ cells (**E**), and FOXP3^−^CD4^+^ cells (**F**). Box plots showing the difference in NND and Z score between NDMM and MGUS for CD8^+^ cells (**G**) and BLIMP1^+^ cells (**H**), and between male and female for BLIMP1^+^ cells (**I**). The unit of NND is μmol/L. The Z score shows the significance of the difference between the NND distribution for a given cell type from a complete spatial random distribution and the observed NND (Supplementary Materials and Methods).

Next, we asked whether the spatial proximity between immune cells and BLIMP1^+^ plasma cells differed according to disease state and treatment. To demonstrate that the spatial analysis result is not dependent on the distance threshold chosen, cell proximity was calculated for a range of distances with the maximum distance set at the cell-cell communication distance of 250 μm (30, 50, 100, 150, 200, 250 μm; refs. 33, 34). Cell proximity data were corrected for cell abundance (Supplementary Materials and Methods; Supplementary Fig. S6A–S6D). The number of FOXP3^+^CD4^+^ cells in proximity to FOXP3^−^CD4^+^ cells decreased at posttreatment compared with the paired diagnostic samples (BH corrected *P* = 0.023 for *r* = 30–250 μm; Supplementary Fig. S7A). However, the number of FOXP3^+^CD4^+^ cells in proximity to CD8^+^ cells was not different between NDMM samples and paired posttreatment samples (BH corrected *P* > 0.05 for *r* = 30–250 μm; Supplementary Fig. S7B). The number of BLIMP1^+^ cells in proximity to CD8^+^ and CD4^+^ cells significantly reduced after treatment (BH corrected *P* < 0.05 for *r* = 30–250 μm; Fig. 6A and Supplementary Fig. S7C, respectively), indicating a significant change in the immune microenvironment posttreatment. However, the number of FOXP3^+^CD4^+^ cells in proximity to FOXP3^−^CD4^+^ and CD8^+^ cells and the number of BLIMP1^+^ cells in proximity to CD4^+^ cells was not different between NDMM and MGUS samples (Supplementary Fig. S7D–S7F). Interestingly, despite similar cell density, the number of BLIMP1^+^ cells in proximity to CD8^+^ cells in MGUS samples was significantly lower than in NDMM samples (BH corrected *P* = 0.036 for *r* = 30–250 μm, Fig. 6B and C), which may indicate variability in antitumor immune activity in the precursor stage compared with the malignant stage.

### Significant spatial clustering of CD8^+^ cells in NDMM samples compared with posttreatment

We next asked how cells distribute within the BM tissues; do they display a spatially dispersed or clustered pattern? To identify the spatial pattern of a specific cell type, we compared the observed NND with the spatial randomness of the cell type within the tissue section (Supplementary Materials and Methods). In most MGUS, NDMM, and posttreatment samples, we observed clustered patterns (Z-score < −1.96) of CD8^+^, BLIMP1^+^ and FOXP3^−^CD4^+^ cells compared with spatial randomness but not for FOXP3^+^CD4^+^ cells (Fig. 6D–H; Supplementary Fig. S8A–S8C). The degree of clustering of CD8^+^ cells in the NDMM was significantly higher at diagnosis than in posttreatment samples (*P* = 0.027, Fig. 6D) but not compared with MGUS samples (*P* = 0.514, Fig. 6G). There was a trend toward increased clustering of BLIMP1^+^ cells in the NDMM samples compared with their paired posttreatment and with MGUS samples (*P* = 0.065 and *P* = 0.06, Fig. 6B and H, respectively). The degree of clustering of BLIMP1^+^ cells in female samples was significantly higher than in male patients (*P* = 0.039, Fig. 6I) but not different between age groups (Supplementary Fig. S8D).

### High accuracy achieved in the validation cohort

The validation cohort contained nine NDMM and paired posttreatment BM samples (*n* = 18) obtained from different hospitals and were stained with MIHC panel 2 using a different Leica Bond RX^m^ autostainer. All samples had a tissue area of above 1.1 × 10^6^ μm^2^, a threshold set for analysis inclusion. They also underwent color normalization before analysis (Supplementary Fig. S9A and S9B). To evaluate the performance of our model on this cohort, 4,857 single-cell annotations (BLIMP1 = 2330, CD4 = 1589, CD8 = 938) and tissue segmentation (e.g., fat, bone, blood) annotations in 54 regions of interest were made on 10 samples. Despite possible variations from tissue processing and staining, MoSaicNet was able to achieve an AUC value of 0.97, 95% CI (0.974–0.978) taking all classes into account (Supplementary Table S11; Supplementary Fig. S10). In addition, each class had a mean AUC of >0.94, reaching an overall accuracy of 0.949, 95% CI (0.946–0.953).

Of the 4,487 superpixels, 227 superpixels were misclassified. Most of the misclassified superpixels were bone being misclassified as blood (65 superpixels), followed by blood being misclassified as tissue (51 superpixels). Taking all classes together, the overall precision value was 0.947, 95% CI (0.942–0.95), the recall value was 0.938, 95% CI (0.933–0.942) and the F1-score was 0.942, 95% CI (0.938–0.945; Supplementary Table S11).

When evaluating the performance of AwareNet in the validation cohort, the single-cell classifier achieved an AUC value of 0.987, 95% CI (0.985–0.988) for BLIMP1^+^ cells, 0.988, 95% CI (0.986–0.989) for CD4 and 0.979, 95% CI (0.973–0.977) for CD8 (Supplementary Fig. S11A–S11C; Supplementary Table S12). The overall accuracy was 0.905, 95% CI (0.901–0.909). Of the 4,857 cells, 441 cells were misclassified. 192 CD8^+^ cells were misclassified as CD4^+^ cells and 103 BLIMP1^+^ cells were misclassified as CD4^+^ cells. Nevertheless, high F1-scores were noted across all three cell types: 0.944, 95% CI (0.94–0.95) for BLIMP1, 0.897, 95% CI (0.89–0.90) for CD4 and 0.814, 95% CI (0.80–0.82) for CD8, with a combined F1-score of 0.885, 95% CI (0.88–0.89; Supplementary Table S12). AUC-PR for all cell types were >0.91 and the Matthew correlation coefficient was 0.85 for this cohort (Supplementary Fig. S11D).

Furthermore, quantitative and spatial analysis of the validation cohort revealed similar findings to the original dataset. As in the original dataset, NDMM samples had significantly higher BLIMP1^+^ cell density (*P* = 0.004, Supplementary Fig. S12A, S12B, and S13A) than posttreatment samples in the validation cohort. Similarly, CD4^+^ T-cell densities were not significantly different between the two groups (*P* = 0.91; Supplementary Fig. S13B). CD8^+^ T-cell densities also did not differ significantly (*P* = 0.82; Supplementary Fig. S13C), a finding at variance with our discovery cohort, this could be due to the small sample size. Spatial analysis demonstrated significantly lower numbers of BLIMP1^+^ cells in proximity to CD4^+^ as well as CD8^+^ T cells in the posttreatment group, in concordance with the original dataset (BH corrected, *P* = 0.003, *r* = 30–250 μm; Supplementary Fig. S14A and S14B).

### *Post hoc* analysis for training dataset sample size calculation

To estimate the sample size needed to train AwareNet and MoSaicNet, we evaluated the performance of these models using different sample sizes and displayed this as learning curves (Supplementary Materials and Methods; Supplementary Fig. S15A and S15B). For AwareNet, using only 40% of the training data, we achieved an F1-score of 0.973 compared with 0.98 when using 100% of the training data (Supplementary Fig. S15A). Thus, by reducing the number of required annotations by about 60%, AwareNet could achieve comparable performance to the model trained on the whole dataset. For MoSaicNet, the model showed the highest performance when trained on 80% of the data, achieving an F1-score of 0.932 compared with a model trained on 100% of the data, with a gap of about 1% (Supplementary Fig. S15B).

## Discussion

Myeloma, like many other blood cancers, initiates and evolves in the BM. The BM ecological niche is highly organized, where hemopoietic, including immune cells, osteoblasts, osteoclasts, adipocytes, and other cells interact and coevolve with neoplastic cells (35, 36). The BM milieu and its architectural pattern are, therefore, crucial to the decoding of neoplasm evolution for many blood cancers. Analysis of the intact BM niche has been limited in the past, both due to the difficulty in preserving epitopes and nucleic acid during the processing of BM trephines and the lack of specialized computational methods that are capable of removing sample artefacts and dissecting BM components.

Here, we demonstrate that, through the generation of carefully preserved BM trephine tissue sections and the development of spatial histology methods based on deep learning and spatial statistics, new biological insights on multiple myeloma neoplastic progression and treatment response can be derived. The spatial architecture of multiple myeloma BM was interrogated by establishing fully automated computational pipelines to analyze immune cells’ spatial topography, bone texture heterogeneity and thickness, in addition to the changes in tumor load and BM components during neoplastic progression and treatment. Previously, spatial interactions of stromal components in BM using three-dimensional microscopy in a mouse model (37) and spatial interactions of BM adipose tissue and hematopoietic stem cells in rhesus macaques were studied (38). To the best of our knowledge, this is the first study to use spatial histology based on deep learning to discover spatial cellular topologies and architectural patterns in human BM trephine samples that inform changes in disease status in multiple myeloma. This is in contrast to the many machine learning methods available for BM aspirate derived cell suspensions for cell counts and marrow evaluation (18, 39). Methods developed in our study may impact the study of many other diseases by unlocking the potential of deep learning and spatial tissue architecture, thus generating new insights from routine BM trephine samples.

BM trephine tissue is a mosaic landscape of blood, bone, cellular tissue, and fat. To dissect the complex mosaic tissue microenvironment into individual components in MIHC images, MoSaicNet was developed. Instead of a standard application of CNNs to generate patch-level (40) or pixel-level classification (30, 41), MoSaicNet can efficiently define the highly irregular tissue component boundary without requiring large amounts of expert annotation training, thus combining the best of two approaches. Patch-based approaches use rigid image patches as units for classification tasks, requiring fewer annotations but cannot generate a detailed mapping of the tissue. In comparison, pixel-based algorithms such as U-Net (30) or Micro-Net (41) generate detailed contours, but such algorithms often require large amounts of training data. MoSaicNet combines a machine learning–based approach, superpixel segmentation, and deep learning classification to efficiently map out the multiple myeloma BM tissue landscape using superpixels as spatial units, classifying them into cellular components, blood, bone, fat, and background.

Building on MoSaicNet, a new autoencoder-based approach was developed to study bone physiology. This was inspired by the potential role of bone and related cells, such as osteoblasts and osteoclasts, in regulating BM remodeling (14, 42) and multiple myeloma dormancy and proliferation (43). Autoencoder is an effective method for dimension reduction and denoising. Here we demonstrated its value in bone texture heterogeneity analysis, using feature extraction based on autoencoder and unsupervised clustering of the bone superpixels. We observed that the amount of bone in the biopsies taken posttreatment was greater than those taken at diagnosis, reflecting the destructive effect of multiple myeloma tumor cells on bone. The bone density of NDMM samples was also more heterogeneous when compared with matched posttreatment samples, again reflecting an effect of the disease process on bone physiology that occurs in a spatially heterogeneous manner (44). Moreover, a novel method was developed to study bone thickness using distance transform and topological analysis. In agreement with the bone trabecular surface analysis on lymphoid cancer samples (12), bone% and bone thickness showed a decreasing pattern with ageing but was not different between male and female samples. Taken together, our data indicate that bone analytical methods may be useful for the study of bone degeneration during multiple myeloma progression and treatment, and bone heterogeneity may be a useful marker for disease activity.

Subsequently, AwareNet, developed specifically to identify rare immune cell types, enabled us to dissect the hematopoietic ecosystem of BM in the context of multiple myeloma. Deep learning models are often sensitive to class imbalance, resulting in lower accuracy in detecting rare cell types such as FOXP3^+^CD4^+^ Tregs in our samples. To resolve this, cell segmentation–based spatial cell weighting was proposed (30, 45). AwareNet extends cell segmentation–based spatial cell weighting (30, 45) by using cell identification instead of segmentation, which is less costly. Furthermore, giving a higher attention score to rare cell types improved the detection of rare cell types compared with U-Net (30) and CONCORDe-Net (13).

Using AwareNet, we observed a reduction in the density of BLIMP1^+^ tumor cells, and of the immune cell subsets, CD8 and Tregs in posttreatment BM, compared with diagnostic samples from paired NDMM. While the reduction in tumor cell density is expected, the decrease in immune cell subsets may suggest an alteration in immune function, such as antitumor responses. Several studies have reported on the changes in frequency or proportion of T-cell subsets in posttreatment BM or blood. However, all these studies have hitherto studied BM aspirate samples and assessed immune cell subsets as a percentage of the CD138-negative fraction of mononuclear cells, while our study quantified cell density as a function of tissue surface area. Thus, although we ourselves have reported an increase in CD8^+^ T cells as a fraction of CD3^+^ cells in posttreatment BM aspirates compared with pretreatment samples (46), it is not possible to directly compare these data. Tregs have attracted a great deal of attention in multiple myeloma, and most studies, including our previous work in BM aspirates, concur in reporting an increased abundance of these cells in patients with multiple myeloma compared with healthy controls (11, 47, 48). Hence, our observation in this study of a greater density of Tregs in NDMM samples compared with posttreatment samples is consistent with previous studies (49). On the other hand, our observation that the density of CD8^+^ cells falls following treatment may be at odds with studies using aspirate samples, for the reasons described above, as well as variation in sampling time and site, but the actual treatments received, and type of transplant are also likely to influence the results (5, 6, 9). Our previous work on BM aspirates found no difference in the actual frequency of Tregs between pretreatment and posttreatment (46).

Importantly, new insights were derived from the topological analysis between (9) plasma cells and immune T cells. In solid tumors such as estrogen receptor–positive breast (50) and lung tumors (34), spatial scores were found to be more prognostic than cell counts. In multiple myeloma, however, the spatial relationship of cells and their prognostic value have remained unexplored. Our approaches control for cell abundance and take into account the local tissue architecture and cell distribution. Interestingly, the number of BLIMP1^+^ cells in spatial proximity with CD8^+^ cells was significantly greater in diagnostic multiple myeloma samples compared with MGUS and posttreatment samples. Given reports of tumor-reactive CD8^+^ T-cell populations in patients with multiple myeloma (51), the proximity of CD8^+^ T cells to tumor cells may represent increased immune activity in multiple myeloma, and the “homing” of CD8^+^ T cells to tumor sites. This is consistent with the clustered pattern of CD8^+^, CD4^+^, and BLIMP1^+^ cells in most cases. We observed a dispersed pattern of FOXP3^+^CD4^+^ Tregs. The expansion of Tregs has been found to contribute to the growth, proliferation, and survival of myeloma plasma cells (9). Thus, the dispersed pattern of Tregs may be a phenotype of expansion, which may promote the invasion and differentiation of multiple myeloma plasma cells.

Accuracy of a deep learning platform often fails when it is applied to a different set of samples with different sample preparation procedures, introducing technical variation (52). BM samples in our validation cohort were collected from different hospitals that may have slightly different tissue processing protocols. They were also stained using a different Leica Bond RX^m^ stainer, resulting in staining variation. With the use of a color normalization step, our deep learning model achieved high overall accuracy with an AUC of >0.9. There was also good concordance in the quantitative and spatial findings between the original and the validation cohort. This suggests that our model could potentially be applied to different datasets after image normalization, maintaining a high performance.

Training machine learning models on limited sample size may result in training bias such as overfitting, impacting the model's performance and generalizability (53). To justify our training sample size, we performed *post hoc* learning curves to evaluate performance of our models against different sample sizes. AwareNet achieved high F1-score of >0.97 when trained on 40% to 100% of the training data, whereas MoSaicNet showed best performance when trained on 80% of the data with a slight drop in performance when trained on 100% of the data. While having more data is believed to generate a better model, adding more heterogeneous data could confuse the model and lead to a reduction in performance (54). This could explain the fluctuation of the model performance in MoSaicNet as the sample size increases. Results from these learning curves suggested that we had an adequate amount of data to train our models.

The limitations of this study include the limited number of samples. More samples are needed to capture the full cellular and noncellular region heterogeneity, and the results should be interpreted with this consideration. Our quantitative and spatial results are likely underpowered, but these are exploratory analyses and as such, there was no prespecified power or sample size. Finally, the MIHC staining contained three parameters. Our next step will be to apply the computational methods developed in this study to more parameters, allowing us to distinguish more immune cell subsets.

To conclude, we demonstrated how spatial and machine learning methods can be used to dissect the mosaic tissue microenvironment of BM trephine samples (MoSaicNet) and accurately identify immune T and multiple myeloma plasma cells (AwareNet). Despite the limited sample size, bone trabeculae morphologic and cell spatial proximity analyses enabled the deep mine of both cellular and noncellular parts of the BM niche. Future works include: (i) adapting MoSaicNet and AwareNet to routinely available hematoxylin and eosin stain of BM trephine samples to further explore bone remodeling; (ii) integrating morphologic and spatial features with molecular features to identify genetic aberrations associated with morphologic or spatial phenotypes in the BM niche; (iii) identifying morphologic and spatial features of progressor and non-progression patients with multiple myeloma precursor conditions (55) to help refine risk models; (iv) exploring the association of bone morphologic features and cellular spatial topography features with patients’ clinical outcomes such as treatment response and survival. Insights generated from this study warrant further validation and investigation in larger cohorts, which is in progress.

## Supplementary Material

Supplementary Data Supplementary methods, tables and figures.

Supplementary Table 10 Supplementary Table 10
