# Supplementary material for: Deep Learning Enables Spatial Mapping of the Mosaic Microenvironment of Myeloma Bone Marrow Trephine Biopsies
Source: Cancer Res. 2024 Jan 11;84(3):493–508. doi: 10.1158/0008-5472.CAN-22-2654 (PMC10831337; doi:10.1158/0008-5472.CAN-22-2654)
Supplement: Supplementary Data — Supplementary methods, tables and figures. [file can-22-2654_supplementary_data_suppsd1.docx]

# Supplementary data

**Sup. Methods**

## Colors of immunohistochemistry staining panels

In MIHC, color is the main discriminant feature used by machine learning algorithms. To avoid collecting new single-cell annotations and training separate models for each panel, we used the same colors for protein expression on the MIHC panels (**Sup. Table 1-2**).

**Deep superpixel classifier**

For a superpixel-based classifier, Konstantinos et al. [1] showed a shallow model performs comparably with VGG[2], InceptionV3[3], and ResNet50[4] models, while reducing the number of parameters by about ten times. Thus, we implemented and trained a custom-designed network to automatically predict the class of the superpixel regions. The custom-designed superpixel classifier CNN consists of convolution layers with {16, 32, 64} neurons followed by two dense layers with {200, 4} neurons. All convolutions were performed using a 3 x 3 filter and followed by a max-pooling layer with a receptive field of 3 x 3 pixels. To minimize the chance of overfitting, a dropout layer (rate=30%) was used between the dense layers. ReLU activation was applied to all layers, but Softmax for the last layer to transform the tensors to probabilities. Parameters were initialized using uniform glorot [5] and optimized using Adam [6] using a learning rate of 10^−4^. We applied categorical cross-entropy loss with class weighting. The model was trained for 500 epochs with patience=50 epochs.

**MoSaicNet pipeline post-processing method**

The output of the classifier is a 4-dimensional class probability vector. The output class/label will be the class with the maximum probability. To convert class labels into segmentation images, all the pixels within the superpixels were assigned the same label/color. The pixels within a superpixel have similar intensity values, and the superpixels are irregularly shaped polygons. To smooth the prediction, we applied a morphological closing operation (Equation 1) with a structuring element (S) of disk with a radius of 20 pixels.

|  | $I' =\left( I \oplus S \right)⊖S$ | (1) |
| --- | --- | --- |

where I, I’, ⊕ and ⊖ are input image, output image, dilation operation, and erosion operation, respectively.

The trained model was applied to WSIs to quantify the amount of cellular tissue in the image. The amount of tissue present in the image was used as quality control for further analysis. Moreover, to speed up the processing time, cell detection and classification models were applied only to the cellular tissue region of the image.

## Bone density representation learning using a convolutional auto-encoder

To understand BM bone texture and structural heterogeneity, the bone region of BM WSI was divided into superpixels and transformed into feature vectors that represent the semantic information. Auto-encoder and patch-based approaches were used to learn the representation of WSIs. However, pixels within a patch might have non-homogenous pixel values. Here, we divided the WSI into superpixels instead of patches and applied a convolutional auto-encoder to learn the lower-dimensional representation of the superpixels.

The convolutional auto-encoder learns a low dimensional representation of superpixels such that it can recover the input from the representation (**Sup. Figure 2B**). The convolutional autoencoder consists of encoder and decoder parts. The encoder transforms the superpixels into a low dimensional latent variable (learned representation) and the decoder reconstructs the input superpixels from the latent variable. The encoder consists of 4 convolutional layers with {8, 16, 32, 64} neurons. Each layer is composed of a 2D convolution layer (filter=3x3, and stride=2), LeakyReLU activation, and batch normalization. The decoder section consists of four layers with a reversed older number of neurons with a transposed 2D convolution layer instead of a 2D convolution layer. We experimented with {2, 8, 32, 64} latent variable dimensions. To optimize model parameters, we applied the mean squared error loss function.

We used a learning rate of 10^-4^ and a batch size of 64. Parameters were initialized using uniform glorot [5] and optimized using Adam [6]. The model was trained for 500 epochs with patience=50 epochs.

**AwareNet post-processing**

To convert the probability map to a binary image, we applied 0.8 as a threshold. To fill holes in the binary image, we applied morphological closing as follows:

|  | $I2=\left( I1 \oplus S \right)⊖S$ | (2) |
| --- | --- | --- |

, where I1, I2, ⊕ and ⊖ are input image, output image, dilation and erosion respectively. S is a disk structuring element of radius 5 pixels. To remove noisy predictions, we excluded objects with an area smaller than 10 pixels^2^. Then, the centre of every object was computed as the centre of a cell. All the thresholds were optimized by maximizing the cell detection F1-score. The cell detection section generates the (x,y) positions of the cells in the image space.

## Visualization of features learned by classifier

A CNN classifier model has feature extraction and classification parts. To visualize the features learned by a classifier model, we extracted the features at the output of the first dense layer of the classifier section, which is a *200-dimensional* vector. To reduce the dimensions of features into 2D without losing information, we applied UMAP.

**Model performance evaluation metrics**

In machine learning, model selection is based on the performance evaluation of validation data. For cell detection and classification, the most commonly used metrics include accuracy, precision, recall, F1-score, and AUC (for classifier). For a binary classifier, given a randomly selected negative (0) and a positive (1), AUC shows the probability that the model prediction for the positive sample will be higher than the negative sample. Considering a binary classifier (positive vs negative), here are terms used in the computation of these metrics:

- True positive (TP): actual value (positive) and model prediction (positive)
- False negative (FN): actual value (positive) and model prediction (negative)
- False positive (FP): actual value (negative) and model prediction (positive)
- True negative (TN): actual value (negative) and model prediction (negative)

The formulas for accuracy, precision, recall, and F1-score are shown below in Equations (3-6).

|  | Accuracy = $\frac{TP + TN}{TP + TN + FP + FN}$ | (3) |
| --- | --- | --- |

|  | Precision = $\frac{TP}{TP + FP}$ | (4) |
| --- | --- | --- |

|  | Recall = $\frac{TP + TN}{TP + FN}$ | (5) |
| --- | --- | --- |

|  | F1-score = 2 $\frac{Recall x Precision}{Recall + Precision}$ | (6) |
| --- | --- | --- |

To estimate the 95% confidence interval (CI) of these above metrics, we applied 1000 bootstraps, with each bootstrap taking 80% of all images using sampling with replacement. A confusion matrix was used to visualize the proportion of correct and incorrect predictions made by the model.

**Cell infiltration patterns: spatial clustering, dispersion, or random**

Quantifying the degree of clustering or dispersion of cells in BM trephine samples is challenging as it can be confounded by the mosaic tissue architecture of the BM trephine (presence of non-cellular tissues, **Sup. Figure 1A**), cell abundance, and the amount of cellular tissue area. In ecology, it was shown that the mean nearest neighbour distance (NND) of all pairs of variables showed the spatial organization of the variables [7]. The NND is the distance from a spatial point to its closest neighbour. Under the null hypothesis, complete spatial randomness (CSR), the distribution of NND is normal [7] (**Figure 2B**). Here, we used the concept of NND and the null hypothesis to identify the infiltration pattern of cells.

Let a given tissue section has *k* cells of type A (e.g BLIMP1+ cells), C={c_i_: i∈{1, 2, 3, k}} . Each cell has an (x, y) position attribute. The NND for cell *c,* the i^th^ element of C, is computed using Equation (7).

|  | ${{NND}_{i} = D}_{c, N(c)} \leq D_{c, j}\forall$ j ∈ C - c | (7) |
| --- | --- | --- |

where D and N(c) are 2-dimensional Euclidean distance and the nearest cell, respectively. Then, the slide level observed NND was computed as mean NND over the k cells (Equation 8).

|  | ${NND}_{obs}= \frac{\sum_{i=1}^{k} {NND}_{i}}{k}$ | (8) |
| --- | --- | --- |

Under a null hypothesis (CSR), the k cells could be at any location in the cellular tissue space (**T**) (**Figure 2B**). Thus, for CSR, C={c_i_: i∈{1, 2, 3, k}}, where (x_i_, y_i_) ∈ T, which is a set of *k* randomly distributed cells across the cellular tissue region. The cellular tissue region was segmented using MoSaicNet (Methods). Then, we computed NND for 300 CSR iterations using Equations 9 and 10. We computed Z-score, to measure the difference between the NND for random distribution of cells and the NND of observed cells pattern (Equation 9) (**Figure 2B**).

|  | $Z-score = \frac{{NND}_{obs} - \mu}{std}$ | (9) |
| --- | --- | --- |

where $\mu$ and std are the mean and standard deviation of NND for the random distribution of cells. Z<-1.96, Z>1.96, and -1.96≤Z≤1.96 indicates a clustered, dispersed and random distribution of observed cells, respectively.

**Staining normalization**

The staining color of digital histopathology images varies according to the scanner type, scanning setting and tissue slide preparation. The variation in staining color is a major challenge in generalisability of digital pathology deep learning models for images obtained in different sites using different scanner or scanning setting [8]. Stain normalization is pre-processing technique in digital pathology that addresses these issues introduced by staining inconsistencies across histopathological images. It plays a crucial role in achieving accurate and generalisable histopathology image analysis.

To address the above challenges different color normalization techniques have developed in computer vision. Some of the techniques manipulate color space transformation. These includes Macenko [9], Vahadane [10] and Reinhard [11]. While others developed deep learning based stain normalization techniques [12][13].

We used Reinhard method due to its simplicity. Reinhard methods uses the target and reference image color or staining statistics to align the color distributions between images, thereby achieving effective color normalization. First, for both target and reference images, the mean and standard deviations of Lab color space are computed. Then, the image statistics from the reference image are mapped to the target image statistics [11].

In our case, the target image was selected from the images that was used during model development making sure it has enough amount of tissue. The reference images are the images from the validation data. Figure 15 shows sample target image and reference images before and after color normalization.

**Sample size estimation using model learning curve**

Expert annotated images are required to train a supervised machine learning-based image classification model. Due to the complexity of the models, the required number of training images is often unknown and not reported [14]. Though it is difficult to know prior to training, there are post-hoc methods such as a learning curve to estimate the sample size required to train a machine learning model [15]. In a learning curve-based sample size estimation, the model is trained using different percentage of the available dataset and evaluated on separately held test data to determine the minimum number of samples needed to train the model without a significant loss of performance compared to a model trained on the whole dataset.

To estimate the learning curve of AwareNet and MoSaicNet, we trained the classification models using different sample sizes (20%, 30%, 40%, 50%, 60%, 70%, 80%, 90% and 100% of the training dataset). The total number of training superpixels and single-cell annotations can be found in **Sup. Table 5** and **Sup. Table** **6**, respectively. The datasets were first shuffled and 10% of the whole dataset was added incrementally to the training data of the previous iteration. For each sample size, the best model was saved during training and the performance of the model was evaluated on the separately held test data to compute the F1-score of the classifier. Since we had multiple classes, for plotting purposes, we computed the macro average of the class-level F1-scores. The learning curve was generated as sample size versus the F1-score curve.

**Supplementary tables**

**Sup. Table 1 | Antibodies used for Leica Bond slide stainer**

| **Antibody** | **Supplier** | **Species** | **Dilution** | **Control** |
| --- | --- | --- | --- | --- |
| BLIMP-1 | CNIO | Mouse | 1:4 | Tonsil |
| CD4 | Novocastra | Mouse | RTU* | Tonsil |
| CD8 | Novocastra | Mouse | RTU* | Tonsil |
| FOXP3 | eBioscience | Rat | 1:100 | Tonsil |

*RTU – ready-to-use from the manufacturer

**Sup. Table 2 | Staining protocols:** CD4 / FOXP3 / CD8 panel

| **Antibody** | **Color** | **Dilution** | **Epitope retrieval protocol** | **Antibody incubation time (min)** | **Post primary incubation time (min)** | **Polymer incubation time (min)** |
| --- | --- | --- | --- | --- | --- | --- |
| CD4 | Brown | RTU | ER2: 20 | 15 | 8 | 8 |
| FOXP3 | Blue | 1:100 | ER1: 10 | 15 | 8 | 8 |
| CD8 | Red | RTU |  | 15 | 8 | 8 |

**Sup. Table 3 | Staining protocols:** CD4 / CD8 / BLIMP1 panel

| **Antibody** | **Color** | **Dilution** | **Epitope retrieval protocol** | **Antibody incubation time (min)** | **Post primary incubation time (min)** | **Polymer incubation time (min)** |
| --- | --- | --- | --- | --- | --- | --- |
| CD4 | Brown | RTU | ER2: 20 | 15 | 8 | 8 |
| BLIMP1 | Blue | 1:4 | ER2: 20 | 15 | 8 | 8 |
| CD8 | Red | RTU | ER2: 20 | 15 | 8 | 8 |

**Sup. Table 4 | Number of manual region segmentation annotation data**

|  | **#Patients** | **#Regions** |
| --- | --- | --- |
| Training | 9 | 126 |
| Validation | 6 | 83 |
| Test | 4 | 51 |

**Sup. Table 5 | Number of superpixels extracted from the human annotation of blood, bone, fat, and tissue regions.**

| **Category** |  | | **Number of superpixels** | | | |
| --- | --- | --- | --- | --- | --- | --- |
|  | **#Patients** | **#Regions** | **Blood** | **Bone** | **Fat** | **Tissue** |
| Training | 9 | 126 | 4560 | 12991 | 10523 | 14338 |
| Validation | 6 | 83 | 1913 | 5642 | 4484 | 6103 |
| Test | 4 | 51 | 1091 | 2275 | 1626 | 4338 |
|  |  |  |  |  |  |  |

**Sup. Table 6 | Distribution of training, validation and testing single-cell annotation data**

|  | **Number of slides** | **CD8^+^** | **FOXP3^-^CD4^+^** | **FOXP3^+^CD4^+^** |
| --- | --- | --- | --- | --- |
| Training | 5 | 2244 | 1000 | 243 |
| Validation | 3 | 1555 | 689 | 140 |
| Test | 3 | 1304 | 692 | 135 |

# Sup. Table 7 | Patient characteristics of the validation cohort

| **Patient characteristics (n=9)** | Patient no. (%) |
| --- | --- |
| **Age at diagnosis** |  |
| Median (range) | 61 (46-67) |
| **Gender** |  |
| Male | 4 (44) |
| **IMWG Cytogenetics risk** |  |
| Standard risk | 7 (78) |
| High risk | 1 (11) |
| Unknown | 1 (11) |
| **IMWG ISS staging** |  |
| I | 4 (44) |
| II | 5 (56) |
| III | 0 (0) |
| **PC % in diagnostic BM biopsy** |  |
| Median (range) | 60 (15-75) |
| **Line of therapy at treatment** |  |
| 1 | 9 (100) |
| **Induction therapy** |  |
| KCRD* | 10 (100) |
| **PC % at D100 BM biopsy post-treatment** |  |
| Median (range) | 0.5% (0-5) |

*K = Carfilzomib, C = cyclophosphamide, R = lenalidomide, D = dexamethasone. PC = plasma cells, BM = bone marrow.

**Sup. Table 8 | MoSaicNet superpixel classifier performance evaluation.** The 95% confidence interval (CI) was computed using 1000 bootstraps. Each bootstrap contained a randomly sampled 80% of the instances.

| **Metric** | **Mean, 95% CI** | **Class** |
| --- | --- | --- |
| AUC | 0.984, [0.983, 0.985] | Bone |
| Precision | 0.88, [0.87, 0.89] | Bone |
| Recall | 0.933, [0.93, 0.94] | Bone |
| F1-score | 0.906, [0.9, 0.91] | Bone |
| AUC | 0.999, [0.999, 0.999] | Blood |
| Precision | 1.0, [1.0, 1.0] | Blood |
| Recall | 0.933, [0.93, 0.94] | Blood |
| F1-score | 0.966, [0.96, 0.97] | Blood |
| AUC | 0.984, [0.983, 0.985] | Tissue |
| Precision | 0.958, [0.95, 0.96] | Tissue |
| Recall | 0.932, [0.93, 0.94] | Tissue |
| F1-score | 0.944, [0.94, 0.95] | Tissue |
| AUC | 0.993, [0.992, 0.994] | Fat |
| Precision | 0.933, [0.93, 0.94] | Fat |
| Recall | 0.954, [0.95, 0.96] | Fat |
| F1-score | 0.943, [0.94, 0.95] | Fat |
| AUC | 0.99, [0.989, 0.991] | All classes |
| Precision | 0.943, [0.94, 0.945] | All classes |
| Recall | 0.938, [0.935, 0.942] | All classes |
| F1-score | 0.94, [0.935, 0.945] | All classes |
|  |  |  |

**Sup. Table 9 | AwareNet single cell classification evaluation metrics.** The 95% confidence interval (CI) was computed using 1000 bootstraps. Each bootstrap contained a randomly sampled 80% of the instances.

| **Metric** | **Mean, 95% CI** | **Class** |
| --- | --- | --- |
| AUC | 0.981, [0.977, 0.989] | FOXP3+CD4+ |
| Precision | 0.857, [0.83, 0.89] | FOXP3+CD4+ |
| Recall | 0.92, [0.9, 0.94] | FOXP3+CD4+ |
| F1-score | 0.887, [0.87, 0.91] | FOXP3+CD4+ |
| AUC | 0.98, [0.976, 0.983] | CD8+ |
| Precision | 0.98, [0.98, 0.98] | CD8+ |
| Recall | 0.98, [0.98, 0.98] | CD8+ |
| F1-score | 0.98, [0.98, 0.98] | CD8+ |
| AUC | 0.98, [0.977, 0.984] | FOXP3-CD4+ |
| Precision | 0.964, [0.96, 0.97] | FOXP3-CD4+ |
| Recall | 0.949, [0.94, 0.96] | FOXP3-CD4+ |
| F1-score | 0.956, [0.95, 0.96] | FOXP3-CD4+ |
| AUC | 0.98, [0.977, 0.984] | All classes |
| Precision | 0.933, [0.923, 0.942] | All classes |
| Recall | 0.949, [0.94, 0.96] | All classes |
| F1-score | 0.941, [0.93, 0.95] | All classes |
|  |  |  |

**Sup. Table 11 | Validation cohort: MoSaicNet superpixel classifier performance evaluation.** The 95% confidence interval (CI) was computed using 1000 bootstraps. Each bootstrap contained a randomly sampled 80% of the instances.

| **Metric** | **Mean, 95% CI** | **Class** |
| --- | --- | --- |
| AUC | 0.949 [0.944, 0.954] | Bone |
| Precision | 0.968 [0.96, 0.97] | Bone |
| Recall | 0.878 [0.87, 0.89] | Bone |
| F1-score | 0.921 [0.91, 0.93] | Bone |
| AUC | 0.971 [0.969, 0.974] | Blood |
| Precision | 0.907 [0.90, 0.92] | Blood |
| Recall | 0.891 [0.88, 0.90] | Blood |
| F1-score | 0.899 [0.89, 0.91] | Blood |
| AUC | 0.982 [0.980, 0.984] | Tissue |
| Precision | 0.952 [0.95, 0.96] | Tissue |
| Recall | 0.981 [0.98, 0.99] | Tissue |
| F1-score | 0.97 [0.96, 0.97] | Tissue |
| AUC | 1.0 [0.999, 1.0] | Fat |
| Precision | 0.959 [0.95,0.96] | Fat |
| Recall | 1.0 [1.0, 1.0] | Fat |
| F1-score | 0.98 [0.98, 0.98] | Fat |
| AUC | 0.976 [0.974, 0.978] | All classes |
| Precision | 0.947 [0.942, 0.950] | All classes |
| Recall | 0.938 [0.9333, 0.942] | All classes |
| F1-score | 0.942 [0.938, 0.945] | All classes |
|  |  |  |

**Sup. Table 12 | Validation cohort: AwareNet single cell classification evaluation metrics.** The 95% confidence interval (CI) was computed using 1000 bootstraps. Each bootstrap contained a randomly sampled 80% of the instances.

| **Metric** | **Mean, 95% CI** | **Class** |
| --- | --- | --- |
| AUC | 0.987 [0.985, 0.988] | BLIMP1+ |
| Precision | 0.959 [0.95, 0.96] | BLIMP1+ |
| Recall | 0.931 [0.93, 0.94] | BLIMP1+ |
| F1-score | 0.944 [0.94, 0.95] | BLIMP1+ |
| AUC | 0.975 [0.973, 0.977] | CD8+ |
| Precision | 0.911 [0.90, 0.92] | CD8+ |
| Recall | 0.735 [0.72. 0.75] | CD8+ |
| F1-score | 0.814 [0.80, 0.82] | CD8+ |
| AUC | 0.988 [0.986, 0.989] | CD4+ |
| Precision | 0.836 [0.83, 0.84] | CD4+ |
| Recall | 0.967 [0.96, 0.97] | CD4+ |
| F1-score | 0.897 [0.89, 0.90] | CD4+ |
| AUC | 0.983 [0.982, 0.984] | All classes |
| Precision | 0.901 [0.898, 0.910] | All classes |
| Recall | 0.878 [0.870, 0.883] | All classes |
| F1-score | 0.885 [0.88, 0.89] | All classes |
|  |  |  |

# Supplementary figures

**
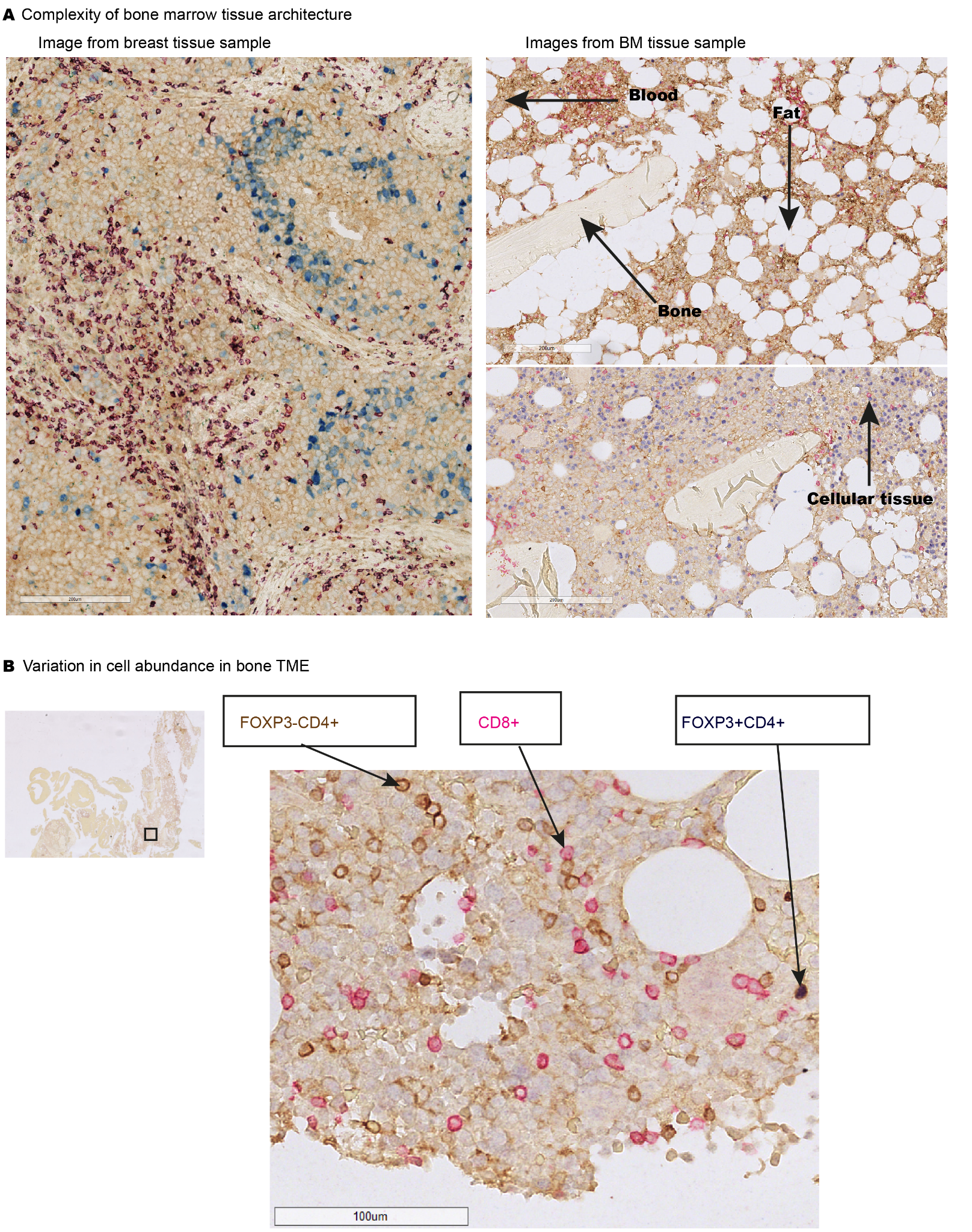
**

**Sup. Figure 1 | Challenges in analysing tissue sections of BM images: A)** Sample images showing the complexity of BM trephine tissue architecture compared to tissue samples from solid tumours (e.g., breast). The image from BM tissue samples is a mosaic of blood, fat, bone, and cellular tissue area.  **B)** The BM is a habitat for rare and abundant cell types. For example, FOXP3^+^CD4^+^ cells are rare compared with CD8^+^ and FOXP3^-^CD4^+^ cells**.**


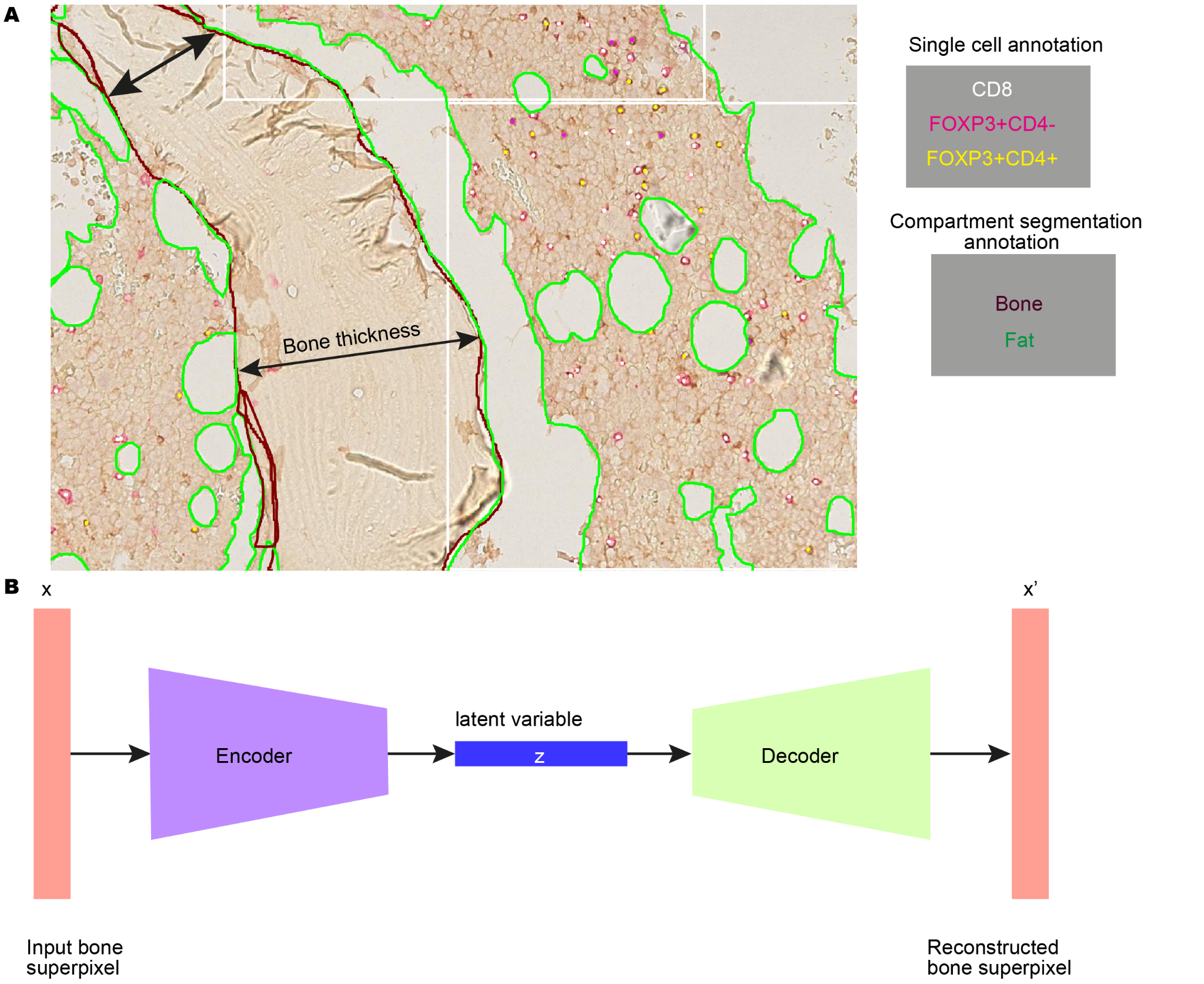


**Sup. Figure 2 | Machine learning algorithms to understand bone physiology:** **A**) Sample image showing expert manual segmentation annotation used to train and validate deep learning models. **B**) Autoencoder architecture that learns low dimensional embedding of bone structure superpixels.

**
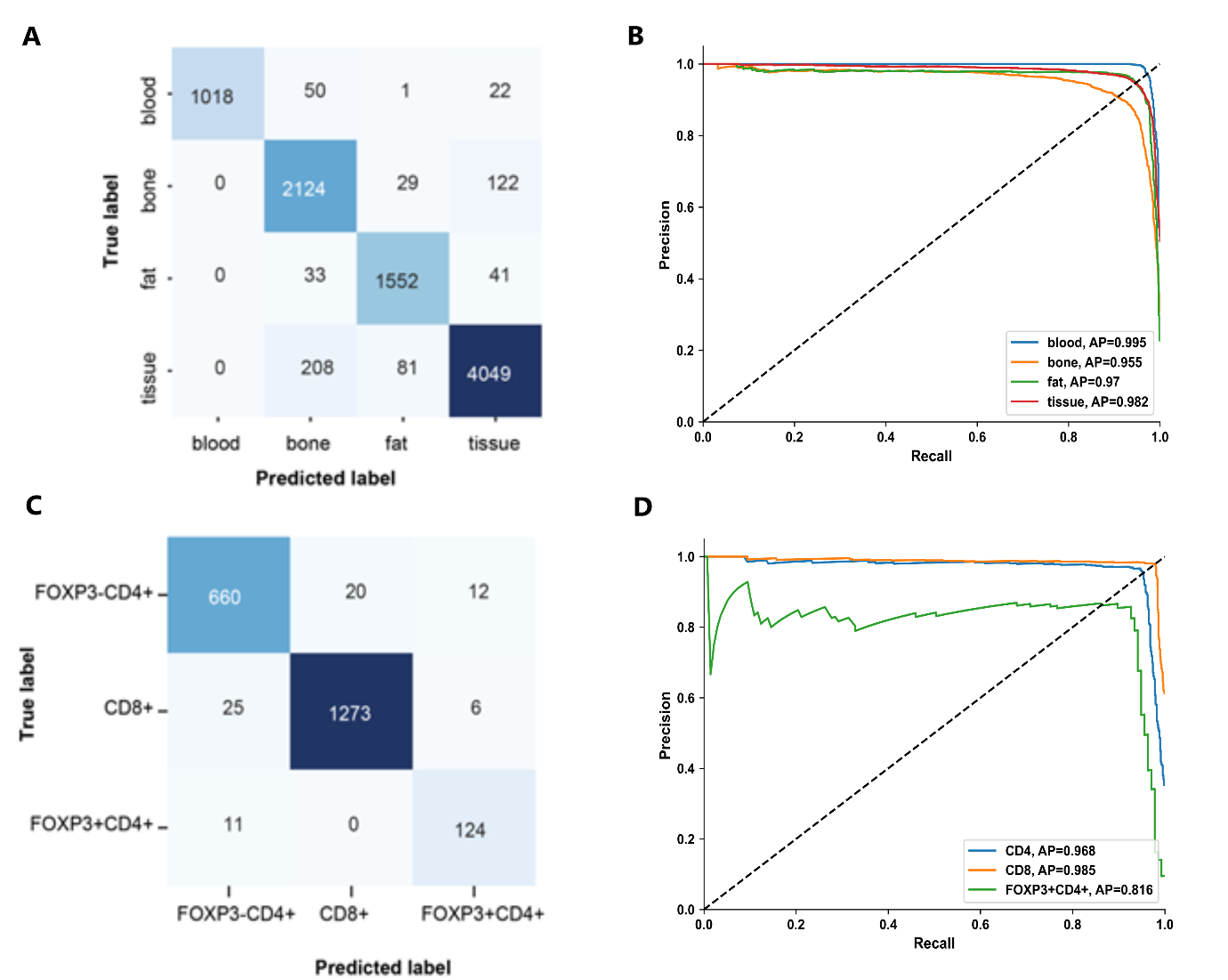
**

**Sup. Figure 3 |** (**A**) Confusion matrix showing classification performance of MoSaicNet. (**B**) Precision-recall curves and Area Under Precision Recall curves (AUC-PR) of MoSaicNet. (**C**) Confusion matrix showing classification performance of AwareNet. (**D**) Precision-recall curves and AUC-PR of AwareNet. The Matthew’s correlation coefficients were 0.91 for MoSaicNet and 0.93 for AwareNet (CD4, CD8, FOXP3 panel).

**
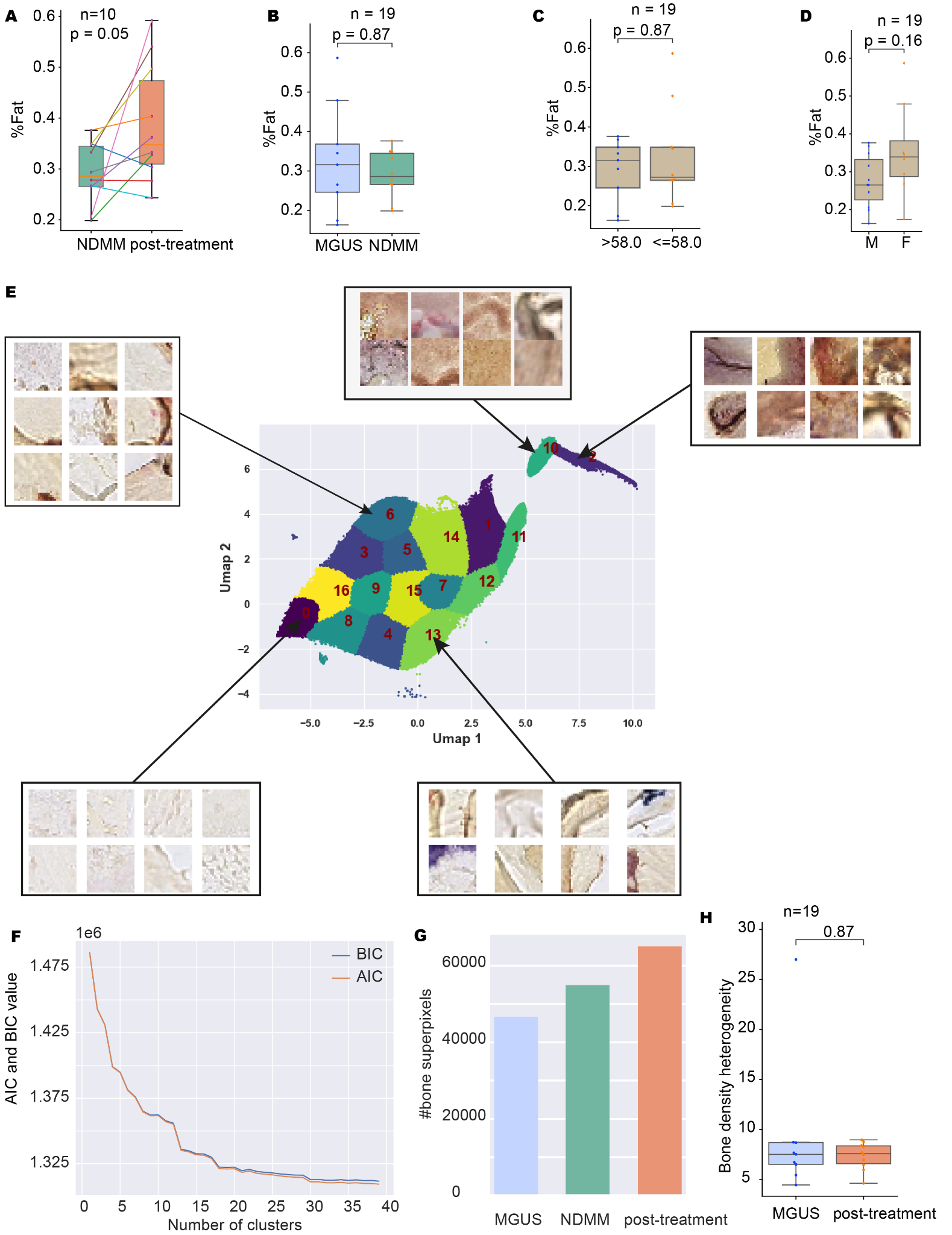
**

**Sup. Figure 4 | Understanding bone physiology from BM tissue samples: A-D**) Boxplots showing the difference in %fat between NDMM and post-treatment (**A**), between MGUS and NDMM (**B**), and between different age groups split by median (**C**) and Gender groups (**D**). **E**) A 2-dimensional mapping of superpixels using MoSaicNet learned 200-dimensional features after dimensionality reduction by Uniform Manifold Approximation and Projection (UMAP). To group similar bone superpixels, we applied Gaussian mixture clustering. The numbers indicate the clustered index. Sample images are displayed for some of the clusters. **F**) Finding the optimal number of clusters using the Akaike information criterion (AIC) and the Bayesian information criterion (BIC). The optimal number of clusters was 19. **G**) Number of bone superpixels extracted from MGUS (9 patients), NDMM (10 patients) and post-treatment (10 patients) samples. **H**) Bone density heterogeneity difference between MGUS and post-treatment samples.


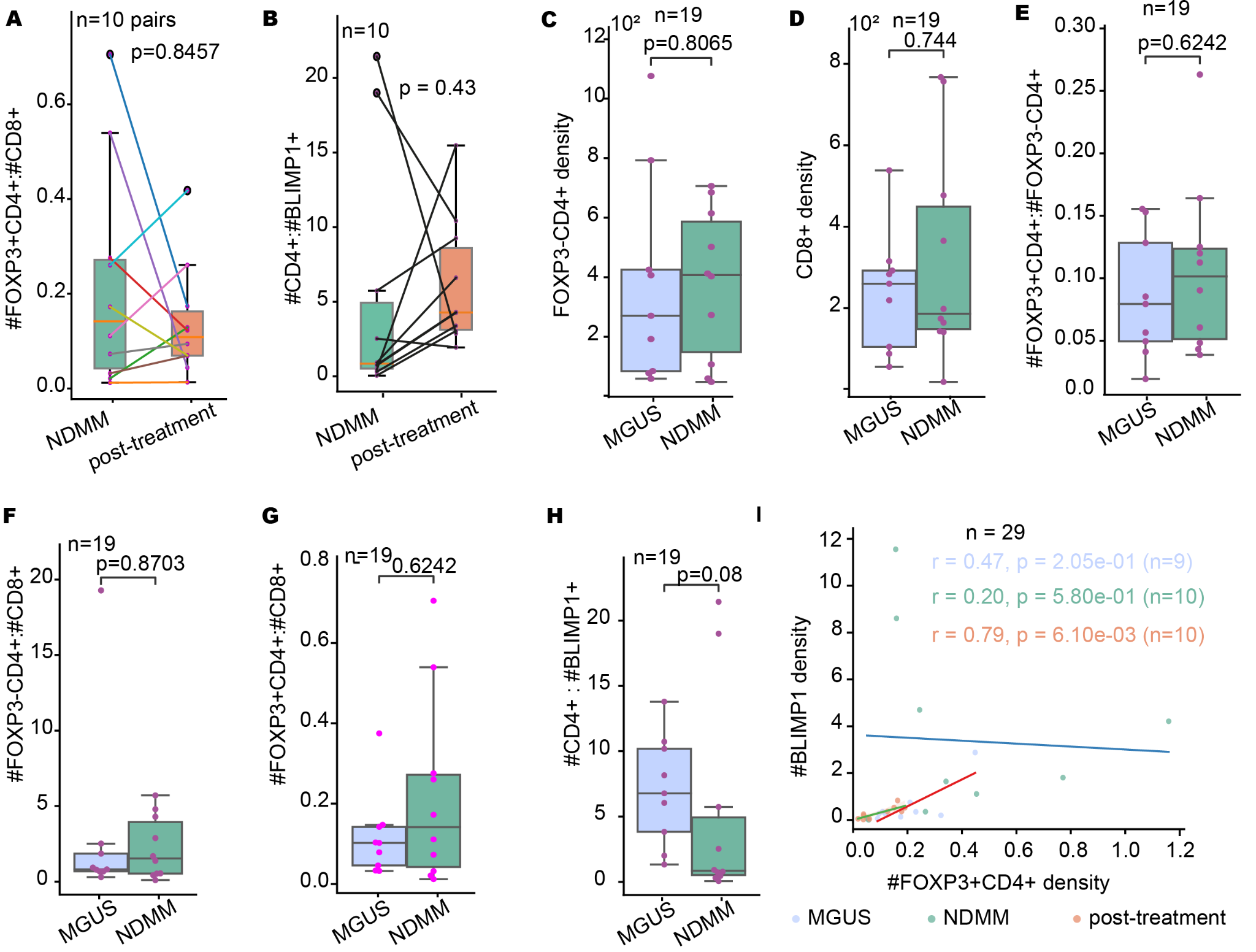


**Sup. Figure 5 | Analysis of cell density and cell ratio: A-B)** Boxplot showing the difference in FOXP3^+^CD4^+^:CD8^+^ ratio (**A**) and CD4^+^:BLIMP1^+^ ratio (**B**) between paired NDMM and post-treatment samples. **C-H**) Boxplots showing the difference in density of FOXP3^-^CD4^+^ (**C**), the density of CD8^+^ (**D**), FOXP3^+^CD4^+^:FOXP3^-^CD4^+^ ratio (**E**), FOXP3^-^CD4^+^: CD8^+^ ratio (**F**), FOXP3^+^CD4^+^: CD8^+^ ratio (**G**), and CD4^+^:BLIMP1^+^ ratio (**H**) between MGUS and NDMM samples. **I**) Correlation between the density of FOXP3^+^CD4^+^ and BLIMP1^+^ cells. The cell density is presented per 1 mm^2^ tissue area.


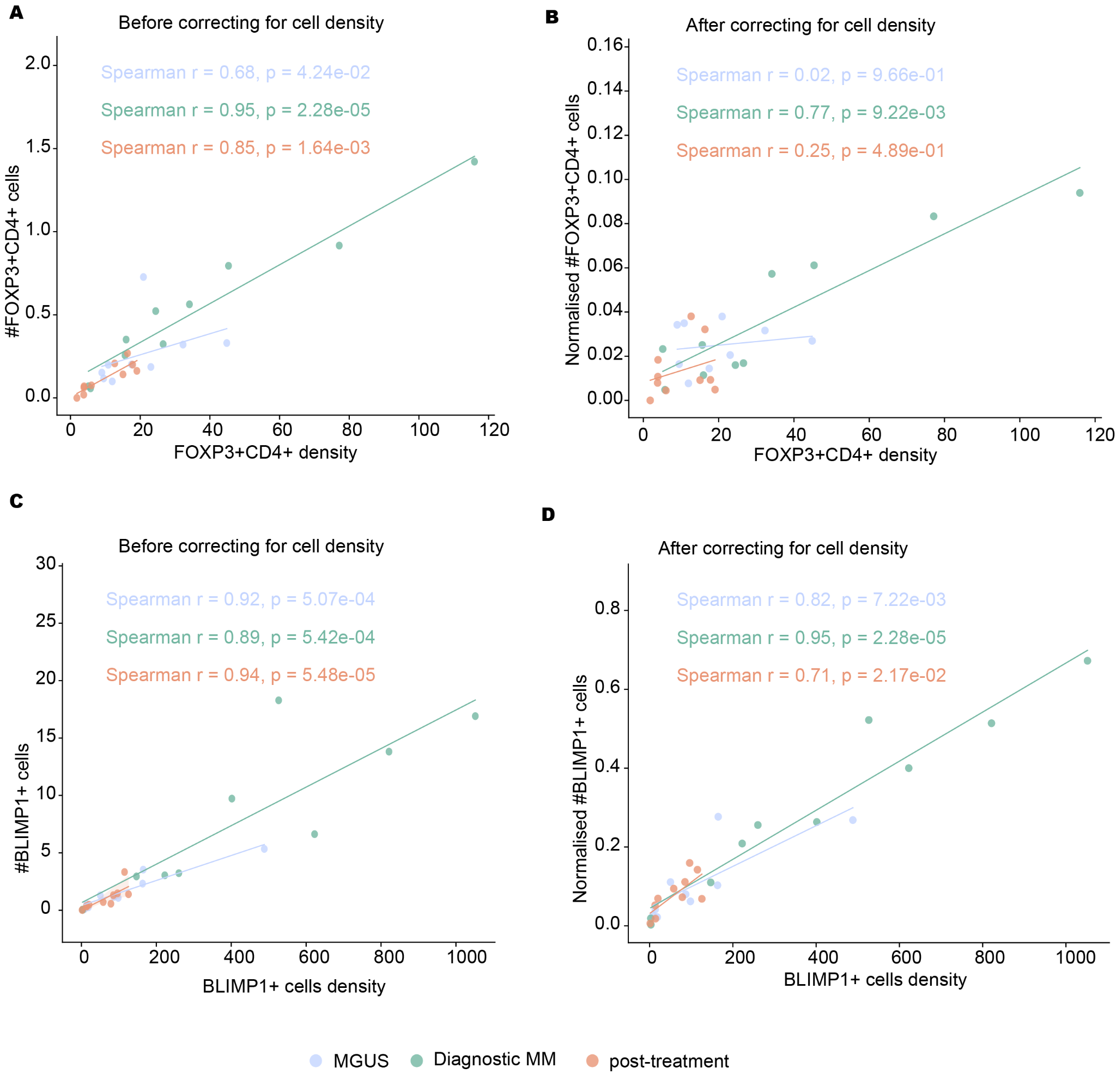


**Sup. Figure 6 | Correlation between cell density and cells' spatial proximity scores:**  Scatter plots showing the correlation between cells' spatial proximity scores before and after correcting for density. **A, B**) Correlation between the number of (#) FOXP3^+^CD4^+^ cells in proximity with CD8^+^ cells before (**A**) and after (**B**) correcting for FOXP3^+^CD4^+^ cell density. After correction, the correlation was reduced in all patient groups. **C, D**) Correlation between the number of (#) BLIMP1^+^ cells in proximity with CD8^+^ cells before (**C**) and after (**D**) correcting for BLIMP1^+^ cell density. After correction, the correlation was reduced in the MGUS and post-treatment groups, but not in the NDMM group. All cell spatial proximity scores were computed for 100μm distance.


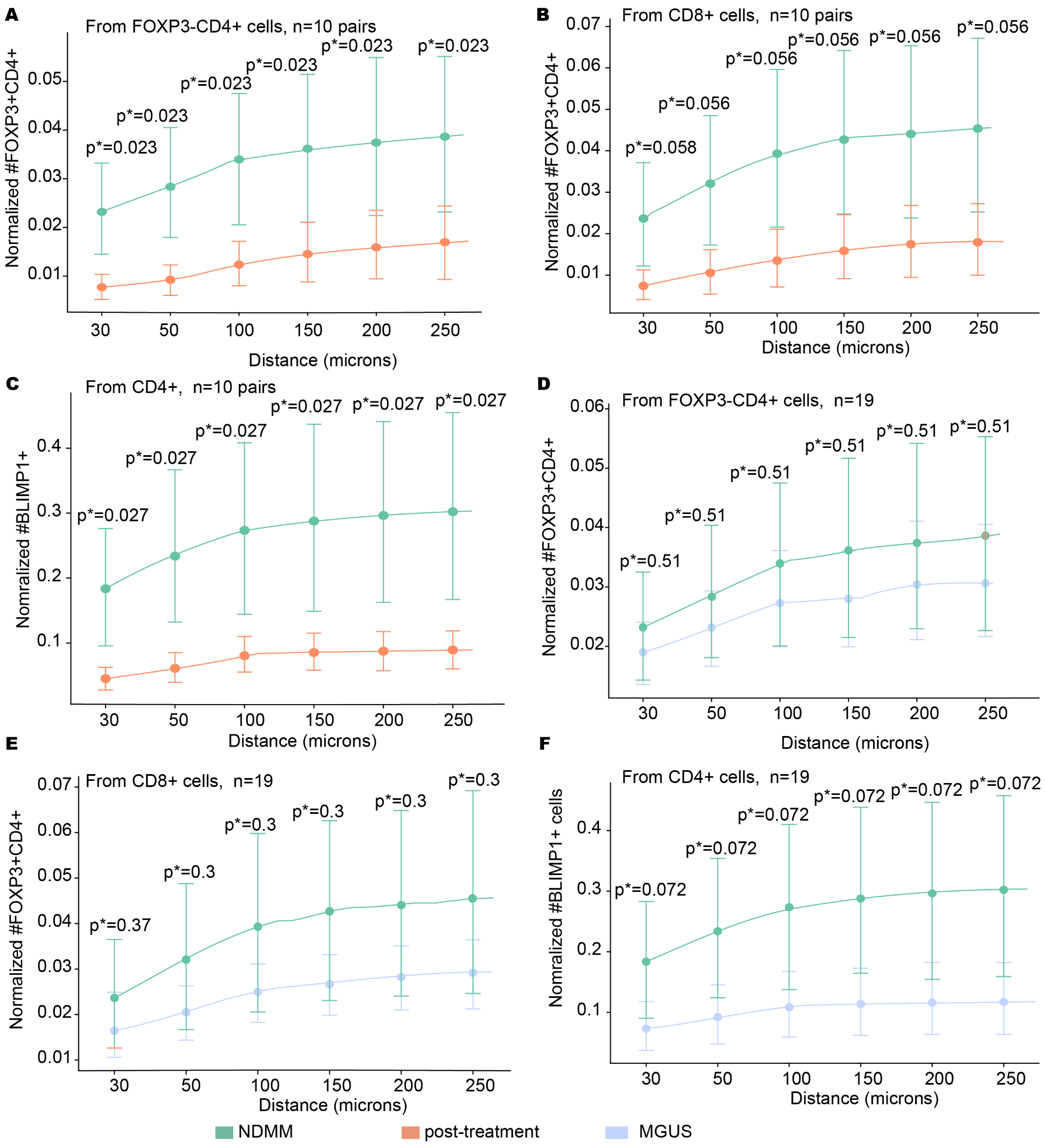


**Sup. Figure 7 | Spatial proximity of immune cells to regulatory T cells and tumour cells:**  **A-B**) Point plots showing the difference in the number of FOXP3^+^CD4^+^ cells within a distance (µm) from FOXP3^-^CD4^+^ cells (**A**) and CD8^+^ cells (**B**) between paired NDMM and post-treatment samples. **C**) Point plot showing the difference in the number of BLIMP1^+^ cells within a distance from CD4^+^ cells between NDMM and post ASCT samples as a function of distance. **D-E**) Point plots showing the difference in the number of FOXP3^+^CD4^+^ cells within a distance (µm) from FOXP3^-^CD4^+^ (**D**) cells and CD8^+^ (**E**) cells among MGUS and NDMM samples. **F)** A point plot showing the difference in the number of BLIMP1^+^ cells within a distance from CD4^+^ cells between MGUS and NDMM samples. In the point plots, the points represent the mean and the bars are 95% confidence intervals, indicating uncertainty.


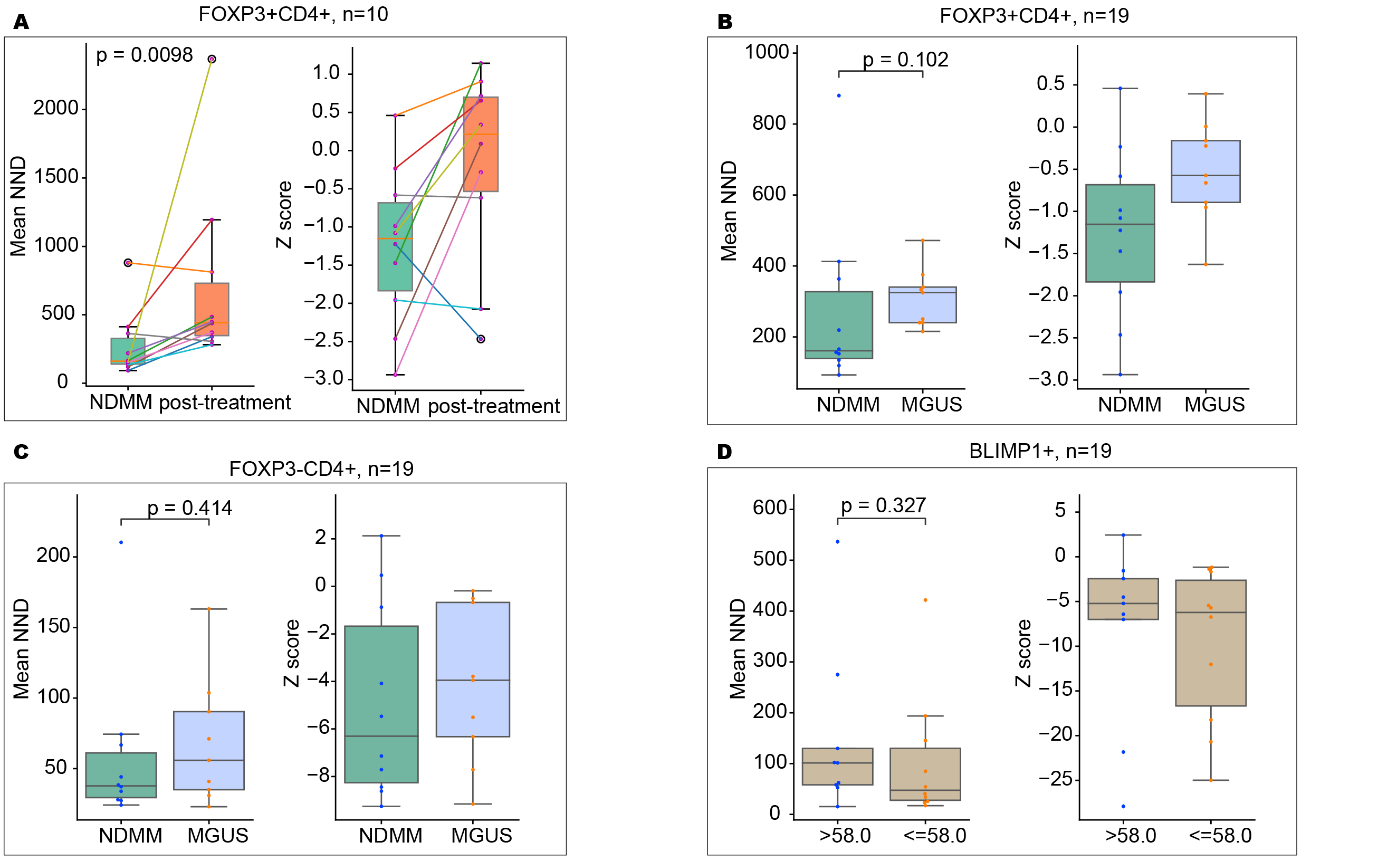


**Sup. Figure 8 | Clustered or dispersed pattern of immune and tumour cells in a BM trephine sample.** Boxplots showing the difference in NND and Z score of FOXP3^+^CD4^+^ cells between NDMM and post-treatment (**A**), FOXP3^+^CD4^+^ cells between NDMM and MGUS (**B**), FOXP3^-^CD4^+^ cells between NDMM and MGUS (**C**), and BLIMP1^+^ cells between age groups (median split) (**D**). The Z score shows the significance of the difference between the NND distribution for a given cell type from a complete spatial random distribution and the observed NND (**Sup. Methods**). The unit of NND is μm.


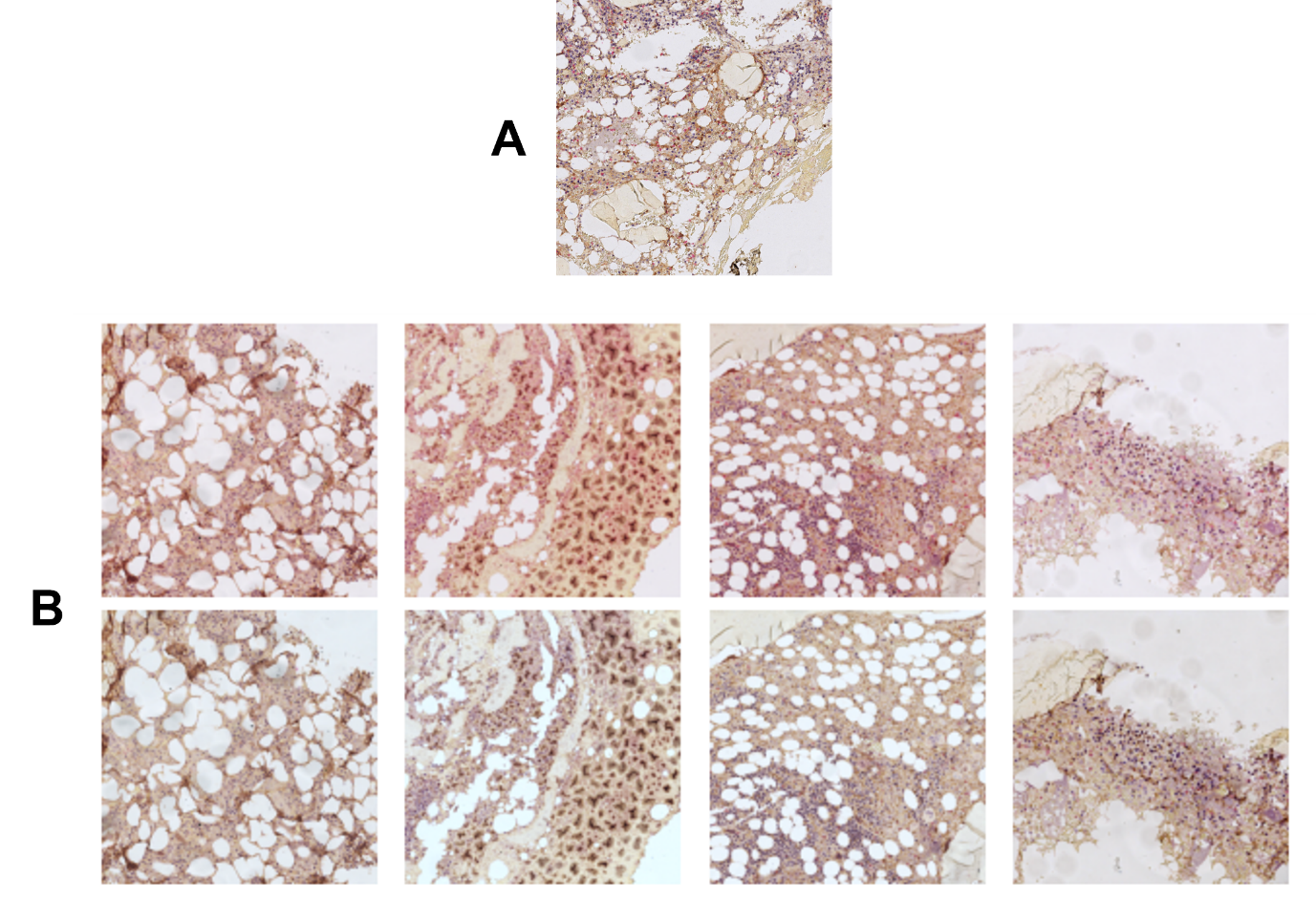


**Sup. Figure 9 | Sample illustrative images showing staining normalization on images from validation cohort images:** A) Target image. B) A list of images before (top row) and after (bottom row) color normalization.

**
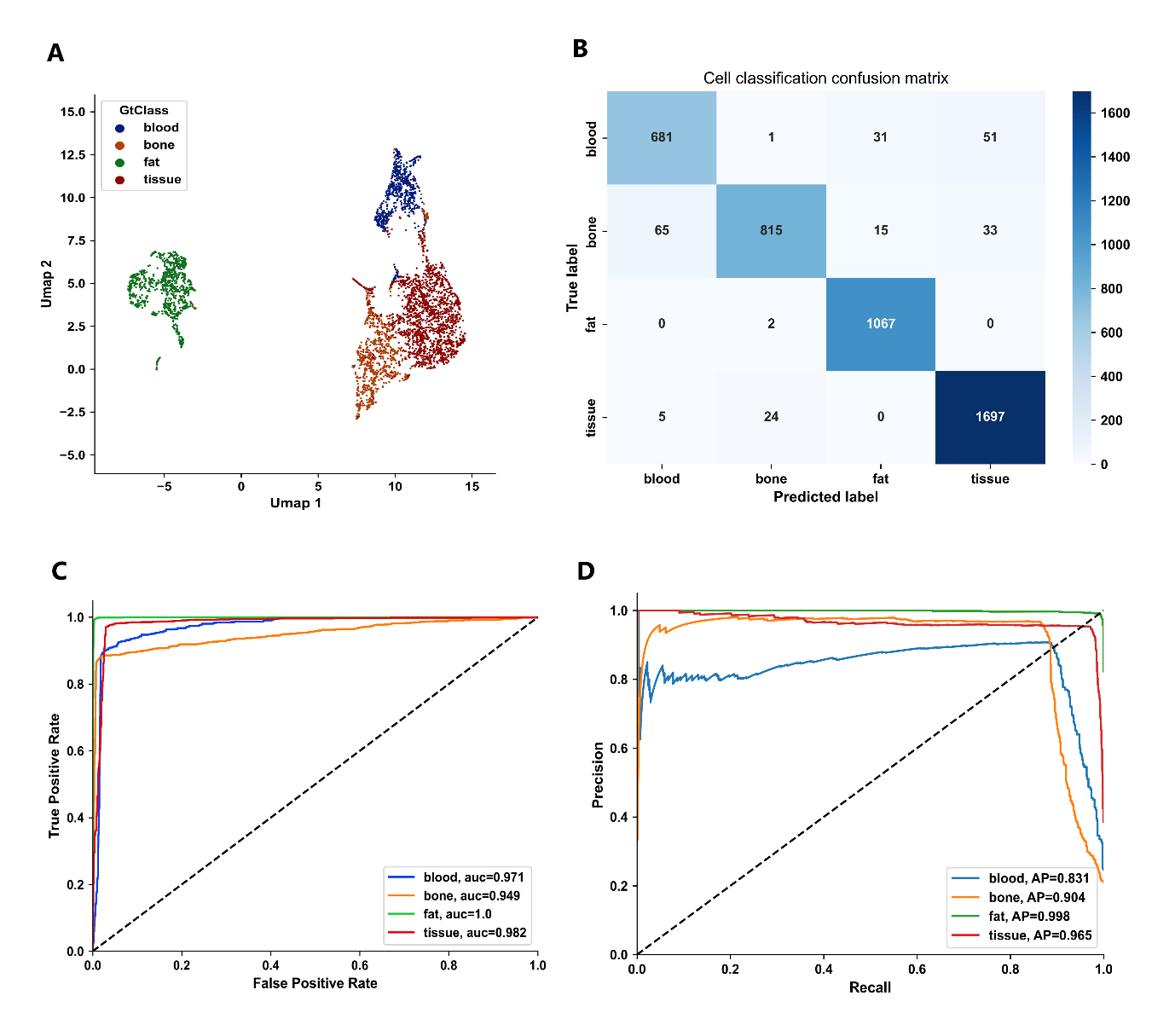
**

**Sup. Figure 10 | Performance evaluation of MoSaicNet on the validation cohort:** (**A**) UMAP features visualization of deep learned features by the superpixel classifier model. (**B**) Confusion matrix showing classification performance. (**C**) The ROC curves and AUC values with regards to different tissue regions. (**D**) Precision-recall curves and Area Under Precision Recall curves (AUC-PR) with regards to different tissue regions. The Matthew’s correlation coefficient was 0.93 for MoSaicNet in this cohort.


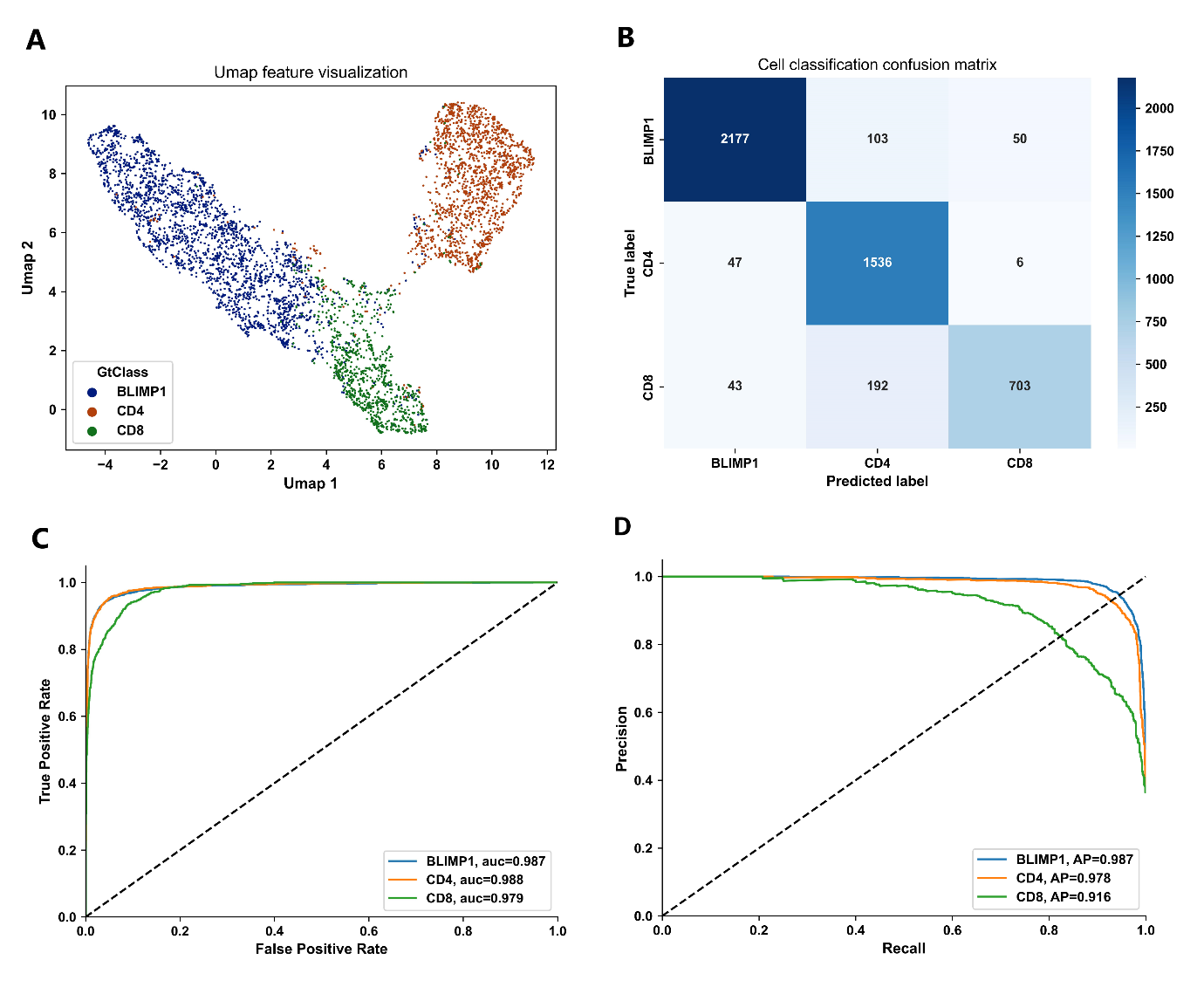


**Sup. Figure 11 | Performance evaluation of AwareNet on the validation cohort:** (**A**) UMAP features visualization of deep learned features by the single-cell classifier CNN. (**B**) Confusion matrix showing classification performance. (**C**) The ROC curves and AUC values with regards to different cell types. (**D**) Precision-recall curves and Area Under Precision Recall curves (AUC-PR) with regards to different cell types. The Matthew’s correlation coefficient was 0.85 for AwareNet in this cohort.


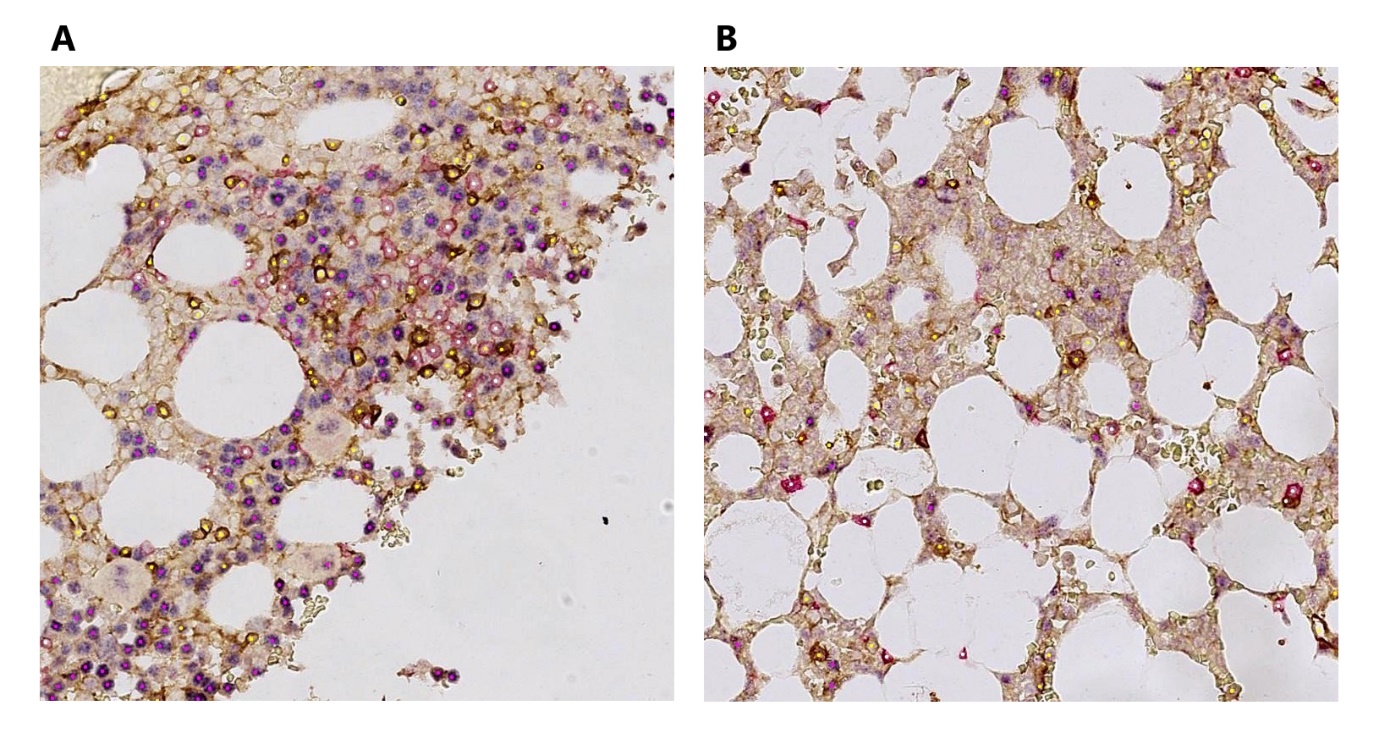


**Sup. Figure 12 | Examples of deep learning output of the validation cohort.** (**A**) is a NDMM sample and (**B**) is a post ASCT sample with visibly fewer BLIMP1^+^ cells (Yellow dot = CD4, white dot = CD8, magenta = BLIMP1).


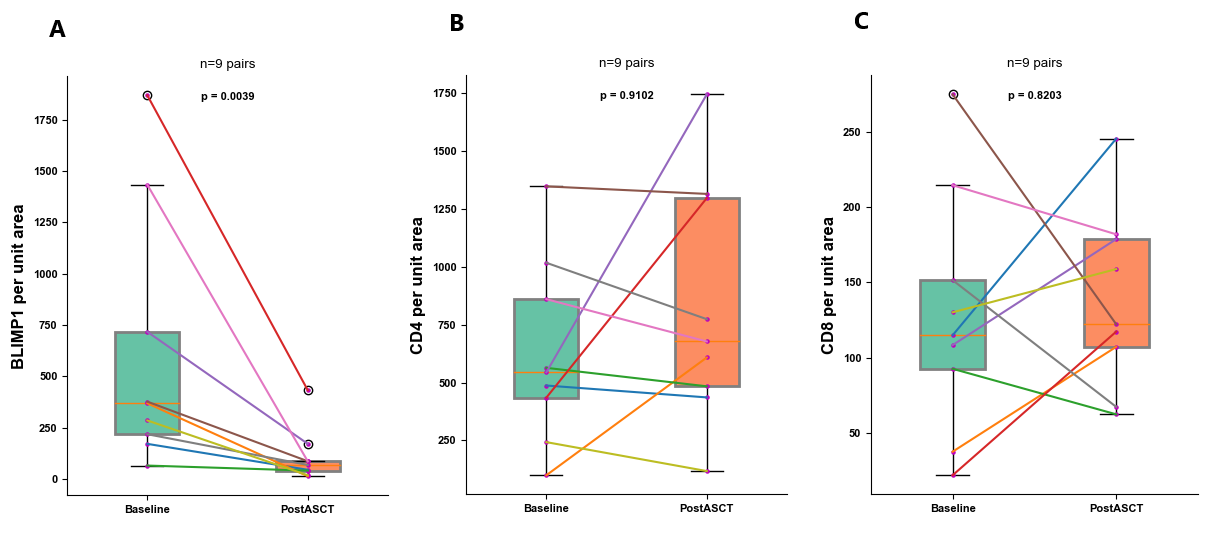


**Sup. Figure 13 | Cell density analysis of the validation cohort:** Boxplots showing the difference in densities of BLIMP1^+^ (**A**), CD4^+^ (**B**) and CD8^+^ (**C**) cells between NDMM and post ASCT samples. The cell density is presented per 1 mm2 tissue area.


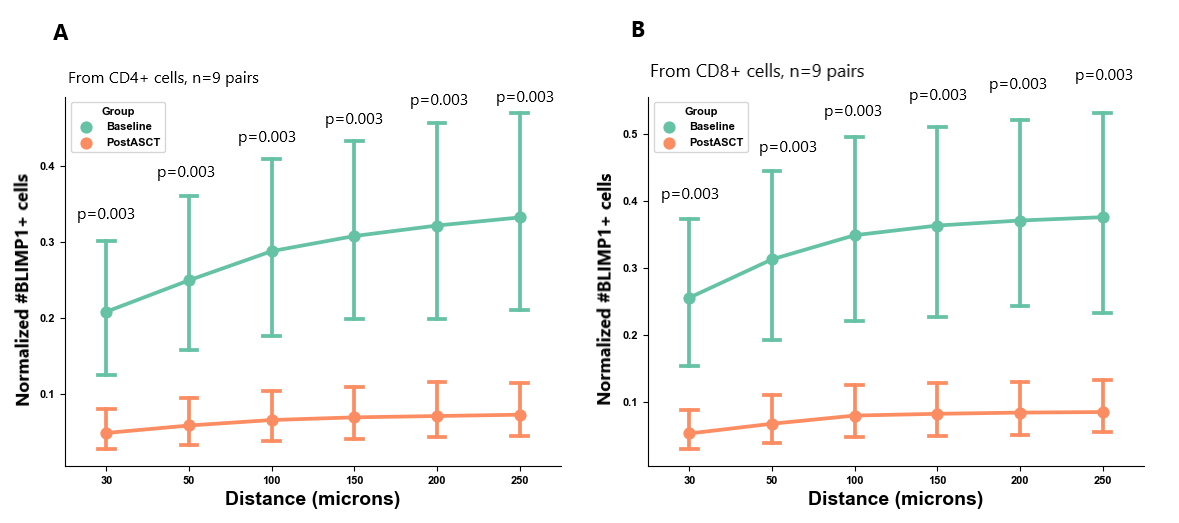


**Sup. Figure 14 | Spatial neighbourhood analysis of the validation cohort:** Point plots showing the difference in the number of BLIMP1^+^ cells within a range of distance (r=30-250 µm) from CD4^+^ cells (**A**) and CD8^+^ cells (**B**) between NDMM and post ASCT samples.

**
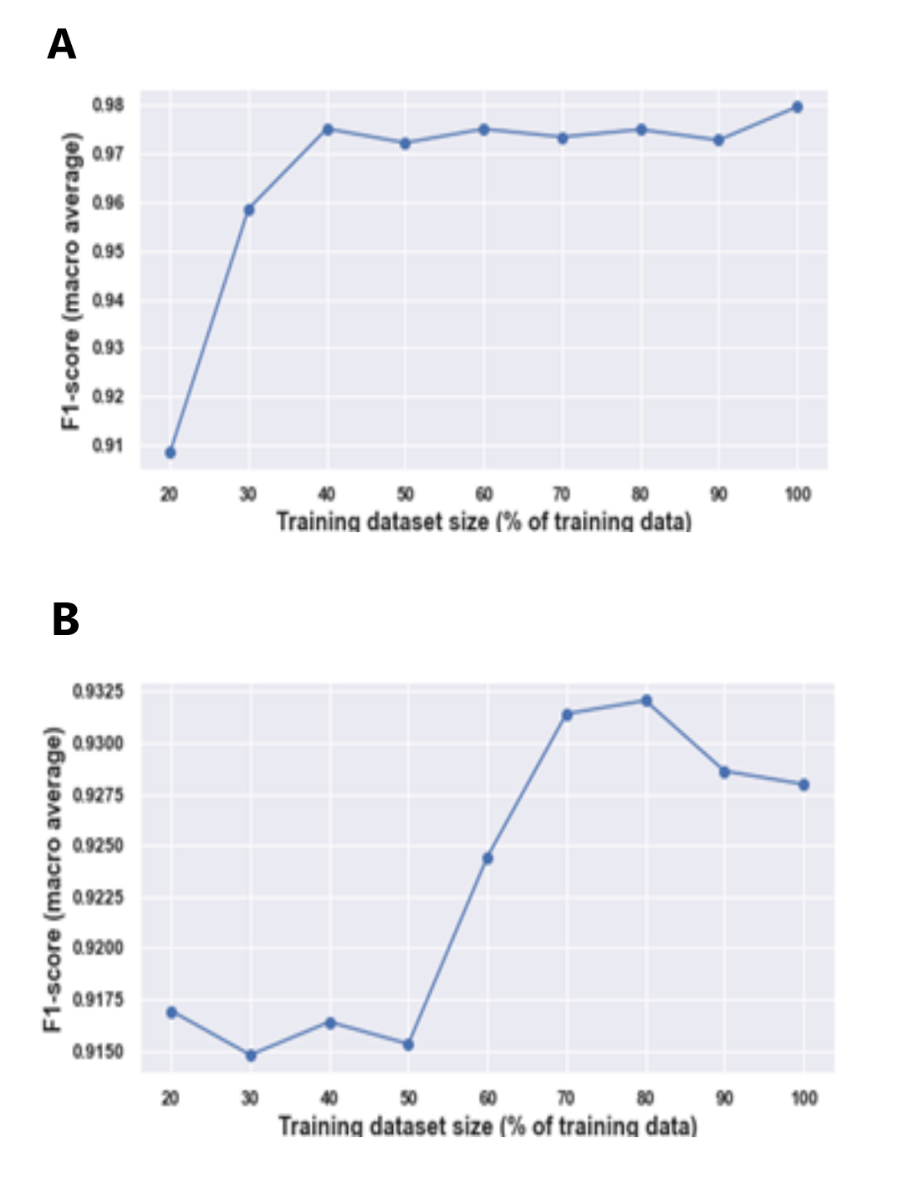
**

**Sup. Figure 15 | Post-hoc analysis for training dataset sample size calculation:** Learning curves showing the performance of AwareNet (**A**) and MoSaicNet (**B**) with different sample sizes. The x-axis shows the percentage (%) of training data and the y-axis is F1-score. The number of training data can be found in Sup. Table 5 and 6.

**References**

[1] Zormpas-Petridis K, Noguera R, Ivankovic DK, Roxanis I, Jamin Y, Yuan Y. SuperHistopath: A Deep Learning Pipeline for Mapping Tumor Heterogeneity on Low-Resolution Whole-Slide Digital Histopathology Images. Front Oncol. 2021;10:586292. doi: 10.3389/fonc.2020.586292. PMID: 33552964; PMCID: PMC7855703.

[2] Simonyan K and Zisserman A. “Very deep convolutional networks for large-scale image recognition,” Sep. 2015. Accessed: Feb. 25, 2021. [Online]. Available: https://www.robots.ox.ac.uk/~vgg/publications/2015/Simonyan15/

[3] Szegedy C, Vanhoucke V, Ioffe S, Shlens J, and Wojna Z, “Rethinking the Inception Architecture for Computer Vision.” Proc. IEEE Comput. Soc. Conf. Comput. Vis. Pattern Recognit., vol. 2016-Decem, pp. 2818–2826, Dec. 2016, doi: 10.1109/CVPR.2016.308.

[4] Kaiming H, Zhang X, Ren S, and Sun J. "Deep residual learning for image recognition." In *Proceedings of the IEEE conference on computer vision and pattern recognition*, pp. 770-778. 2016.

[5] Glorot X and Bengio Y, “Understanding the difficulty of training deep feedforward neural networks,” JMLR Workshop and Conference Proceedings, Mar. 2010. Accessed: Jan. 05, 2021. [Online]. Available: https://proceedings.mlr.press/v9/glorot10a.html

[6] Kingma DP and Ba JL, “Adam: A method for stochastic optimization,” Dec. 2015, Accessed: Jan. 05, 2021. [Online]. Available: https://arxiv.org/abs/1412.6980v9.

[7] Clark PJ and Evans FC. "Distance to nearest neighbor as a measure of spatial relationships in populations." Ecology 35.4 (1954): 445-453.

[8] Michielli N, Caputo A, Scotto M, Mogetta A, Pennisi OAM, Molinari F, Balmativola D, Bosco M, Gambella A, Metovic J, Tota D, Carpenito L, Gasparri P, Salvi M. Stain normalization in digital pathology: Clinical multi-center evaluation of image quality. J Pathol Inform. 2022 Sep 24;13:100145. doi: 10.1016/j.jpi.2022.100145. PMID: 36268060; PMCID: PMC9577129.

[9] Macenko M, Niethammer M, Marron JS, Borland D, Woosley JT, Guan X, Schmitt C, Thomas NE. "A method for normalizing histology slides for quantitative analysis." 2009 IEEE international symposium on biomedical imaging: from nano to macro. IEEE, 2009.

[10] Vahadane A, Peng T, Sethi A, Albarqouni S, Wang L, Baust M, Steiger K, Schlitter AM, Esposito I, Navab N. Structure-Preserving Color Normalization and Sparse Stain Separation for Histological Images. IEEE Trans Med Imaging. 2016 Aug;35(8):1962-71. doi: 10.1109/TMI.2016.2529665. Epub 2016 Apr 27. PMID: 27164577.

[11] Reinhard E, Adhikhmin M, Gooch B, Shirley P. "Color transfer between images." IEEE Computer graphics and applications 21.5 (2001): 34-41.

[12] Gutiérrez Pérez JC, Otero Baguer D, Maass P. StainCUT: Stain Normalization with Contrastive Learning. J Imaging. 2022 Jul 20;8(7):202. doi: 10.3390/jimaging8070202. PMID: 35877646; PMCID: PMC9317097.

[13] Kang H, Luo D, Feng W, Zeng S, Quan T, Hu J, Liu X. StainNet: A Fast and Robust Stain Normalization Network. Front Med (Lausanne). 2021 Nov 5;8:746307. doi: 10.3389/fmed.2021.746307. PMID: 34805215; PMCID: PMC8602577.

[14] Figueroa RL, Zeng-Treitler Q, Kandula S, Ngo LH. Predicting sample size required for classification performance. BMC Med Inform Decis Mak. 2012 Feb 15;12:8. doi: 10.1186/1472-6947-12-8. PMID: 22336388; PMCID: PMC3307431.

[15] Balki I, Amirabadi A, Levman J, Martel AL, Emersic Z, Meden B, Garcia-Pedrero A, Ramirez SC, Kong D, Moody AR, Tyrrell PN. Sample-Size Determination Methodologies for Machine Learning in Medical Imaging Research: A Systematic Review. Can Assoc Radiol J. 2019 Nov;70(4):344-353. doi: 10.1016/j.carj.2019.06.002. Epub 2019 Sep 12. PMID: 31522841.
